# Aberrant Expressions of Co-stimulatory and Co-inhibitory Molecules in Autoimmune Diseases

**DOI:** 10.3389/fimmu.2019.00261

**Published:** 2019-02-20

**Authors:** Weiwei He, Bin Wang, Qian Li, Qiuming Yao, Xi Jia, Ronghua Song, Sheli Li, Jin-an Zhang

**Affiliations:** ^1^Department of Endocrinology, Affiliated Hospital of Yanan Medical University, Yanan, China; ^2^Department of Endocrinology, Jinshan Hospital of Fudan University, Shanghai, China; ^3^Department of Endocrinology and Rheumatology, Shanghai University of Medicine and Health Sciences Affiliated Zhoupu Hospital, Shanghai, China

**Keywords:** autoimmune diseases, co-stimulatory molecules, co-inhibitory molecules, Graves' disease, CD160

## Abstract

Co-signaling molecules include co-stimulatory and co-inhibitory molecules and play important roles in modulating immune responses. The roles of co-signaling molecules in autoimmune diseases have not been clearly defined. We assessed the expressions of co-stimulatory and co-inhibitory molecules in autoimmune diseases through a bioinformatics-based study. By using datasets of whole-genome transcriptome, the expressions of 54 co-stimulatory or co-inhibitory genes in common autoimmune diseases were analyzed using Robust rank aggregation (RRA) method. Nineteen array datasets and 6 RNA-seq datasets were included in the RRA discovery study and RRA validation study, respectively. Significant genes were further validated in several autoimmune diseases including Graves' disease (GD). RRA discovery study suggested that CD160 was the most significant gene aberrantly expressed in autoimmune diseases (Adjusted *P* = 5.9E-12), followed by CD58 (Adjusted *P* = 5.7E-06) and CD244 (Adjusted *P* = 9.5E-05). RRA validation study also identified CD160 as the most significant gene aberrantly expressed in autoimmune diseases (Adjusted *P* = 5.9E-09). We further found that the aberrant expression of CD160 was statistically significant in multiple autoimmune diseases including GD (*P* < 0.05), and CD160 had a moderate role in diagnosing those autoimmune diseases. Flow cytometry confirmed that CD160 was differentially expressed on the surface of CD8^+^ T cells between GD patients and healthy controls (*P* = 0.002), which proved the aberrant expression of CD160 in GD at the protein level. This study suggests that CD160 is the most significant co-signaling gene aberrantly expressed in autoimmune diseases. Treatment strategy targeting CD160-related pathway may be promising for the therapy of autoimmune diseases.

## Introduction

Autoimmune diseases are complex diseases in which aberrant immunity cause immune attacks and serious damage to normal human tissues or organs (1, 2). Despite considerable progress in the studies of both the risk factors and treatment of autoimmune diseases, the mechanisms of most autoimmune diseases remain largely elusive (3–5). Current literature supports both genetic and environmental factors have important roles in the development of autoimmunity (6, 7). Genome-wide association studies have provided deeper insights into the genetic causes of autoimmune diseases (8). Additionally, some environmental factors are important risk factors of autoimmune diseases, such as vitamin D deficiency and infections (6, 9–11). Recent epigenetic studies have further proven the essential role of epigenetics in the pathogeneses of autoimmune diseases (12–14). Nevertheless, the molecular mechanisms underlying the development of autoimmunity are still not clearly defined, and additional studies are needed (15, 16).

It has been well accepted that abnormal interactions between immune cells have crucial roles in the development of autoimmunity (17–19). Co-signaling molecules include co-stimulatory and co-inhibitory molecules and play essential roles in modulating the interactions of immune cells (20, 21). The activation and differentiation of T cells are directed by both the antigen-specific signal and costimulation signal, and T-cell costimulation is a critical part during the induction of T cells-mediated immune response (22). During intracellular contacts, specific recognition between co-signaling molecules can trigger changes in the expressions of functions of downstream elements and thus regulate TCR signals (22). Co-stimulatory molecules can enhance TCR-mediated immune responses and are pivotal in the activation of T cells. Co-inhibitory molecules can inhibit TCR-mediated immune responses, and regulation of T cells-mediated immune responses can be achieved by the expressions of co-inhibitory molecules on B cells, antigen-presenting cells (APCs), or peripheral tissues (23). Therefore, co-stimulatory and co-inhibitory molecules are important controllers of T-cell responses, and a precise balance between these pathways is crucial in preventing the development of autoimmune diseases (24).

T cells have obligatory roles in the pathogeneses of most autoimmune diseases (25, 26). Because T-cell costimulation is a critical part during the induction of T cells-mediated immune response, the roles of co-signaling molecules in autoimmune diseases also have gained much attention (21). There is increasing evidence that some co-signaling molecules have a pivotal role in the pathogeneses of autoimmune diseases, which may contribute to promising therapeutic targets (23, 27). There are multiple co-stimulatory and co-inhibitory pathways, but no study systematically assesses the aberrant expressions of these genes in autoimmune diseases. We thus sought to assess the expressions of co-stimulatory and co-inhibitory molecules in autoimmune diseases through both a bioinformatics-based study and a validation study using clinical samples.

## Methods

### Datasets of Autoimmune Diseases

NIH Gene Expression Omnibus (GEO) database was systematically searched to identify datasets of whole-genome transcriptome of autoimmune diseases, such as systemic lupus erythematosus (SLE), rheumatoid arthritis (RA), inflammatory bowel disease (IBD), juvenile idiopathic arthritis (JIA), type 1 diabetes mellitus (T1DM), autoimmune thyroiditis, Graves' disease (GD), psoriatic arthritis (PsA), Sjögren's syndrome and ankylosing spondylitis. To be included in our study, datasets must meet the following eligibility criteria: (1) Array datasets must contain at least 20 cases and at least 20 controls, while RNA-seq datasets should have at least 5 cases and at least 5 controls; (2) Assessing transcriptome in whole blood or peripheral blood mononuclear cells (PBMCs); (3) Identifying at least 50 differentially expressed genes (DEGs); (4) Raw data or gene expression profiling were available in GEO; (5) Containing the expressions of at least 90% of total co-signaling molecules analyzed in this study. To exclude the possible impact of treatment on the expressions of those co-signaling molecules, we only used data of the first visit for patients with transcriptome data of multiple visits.

### RRA Discovery Study

To integrate the outcomes from multiple datasets, robust rank aggregation (RRA) method was utilized, which is a well-designed tool to analyze data from multiple arrays (28, 29). We firstly performed a RRA discovery study by using data from all array datasets. Both the gene expression matrix and related annotation document for each array dataset were downloaded from GEO database, and microarray probes were then mapped to gene symbols using corresponding annotation document. If multiple probes mapped to the same symbol, the mean value was used. The expression values of 54 co-stimulatory or co-inhibitory genes were subsequently extracted (Supplementary Table 1). Samples of each dataset were categorized into two groups including patients with autoimmune diseases and healthy controls. We uniformly used the log-transformed expression data, and those datasets without logarithmic transformation were firstly log_2_-transformed before calculating DEGs. The data above were then normalized using the “limma” package for R and DEGs were finally determined.

Before RRA analysis, both the up-ranked and down-ranked gene lists in each dataset were generated by their fold changes between cases and controls. The ranked gene lists of all eligible datasets were then finally integrated using “Robust Rank Aggregation” package for R software. *P-*value in the RRA tool indicated the possibility of ranking high in the final gene list, and genes with adjusted *P-*value less than 0.05 were considered as significant genes in the RRA analysis. Subgroup analysis by the type of data (whole blood or PBMCs) and the types of platforms (Illumina or Affymetrix). We also further added a sensitivity analysis by normalizing gene expression values across datasets through the ComBat of SVA R package (30).

### RRA Validation Study

Previous studies have uncovered that array has several disadvantages, such as cross-hybridization risk, and RNA-seq data are highly replicable and can provide a more accurate assessment of gene expression than array (31–34). Therefore, we further performed a RRA validation study by using data from RNA-seq datasets. Raw RNA-seq read count data were analyzed using DESeq2 to identify DEGs (35). Both the up-ranked and down-ranked gene lists of those 54 co-signaling molecules in each dataset were generated by their fold changes between cases and controls. RRA analysis was then performed to integrate the outcomes above. Genes with adjusted *P-*value less than 0.05 were considered as significant genes in the RRA analysis.

### Validation Study in SLE, RA, IBD, and JIA

We compared the expressions of CD160 and CD58 between cases and controls in four RNA-seq datasets including SLE (GSE112087), RA (GSE117769), IBD (GSE112057IBD), and JIA (GSE112057JIA), respectively. The transcripts per million (TPM) values of all genes, which could provide a more accurate measure of the true abundance of mRNA (36, 37), were calculated from the raw read counts. The TPM values of CD160 and CD58 were then extracted and were compared between cases and controls.

### Samples Collection

Blood samples from newly diagnosed GD patients and healthy controls were collected in our hospital. GD was diagnosed by thyroid enlargement and hyperthyroidism, accompanied by increased thyrotropin receptor-stimulating antibody (TRAb). There were 42 GD patients and 36 healthy controls in the study of quantitative real-time PCR (qRT-CPR), and 23 GD patients and 21 healthy controls in the study of flow cytometry. The characteristics of those GD patients and controls were shown in the Supplementary Tables 2, 3. PBMCs from 2 mL EDTA anticoagulated peripheral blood were isolated by density gradient centrifugation using lymphocyte separation medium, and 1 mL TRIzol reagent were then added to store total RNA. PBMCs isolated from 5 mL heparin sodium anticoagulated peripheral blood were used for the study of flow cytometry. The study was approved by the Institute Ethics Committee of Shanghai University of Medicine & Health Sciences Affiliated Zhoupu Hospital, and written informed consent was obtained from all participants in accordance with the Declaration of Helsinki.

### RNA Extraction and qRT-PCR

RNA was extracted with an RNeasy Kit (Qiagen), and complementary DNA (cDNA) was then synthesized from 1 μg RNA. qRT-PCR was performed using SYBR Green PCR Master Mix with a total reaction volume of 15 μl in triplicate. The reactions were performed using Applied Biosystems 7500 Real-Time PCR System. PCR program was as follow: 30 s at 95°C for one cycle, then 40 two-step cycles of 5 s at 95°C and 34 s at 63°C. We performed qRT-PCR using three house-keeping genes as reference including B2M, ACTB (beta-actin) and GAPDH, which are abundantly expressed in the immune cells of peripheral blood (38). Primer sequences were as follows: ACTB fwd, 5′-CAT TGC CGA CAG GAT GCA G-3′; ACTB rev, 5′-CTC GTC ATA CTC CTG CTT GCT G-3′; B2M fwd, 5′-CAT CCA TCC GAC ATT GAA GTT-3′; B2M rev, 5′- ACG GCA GGC ATA CTC ATC TTT-3′; GAPDH fwd, 5′-GGA GCG AGA TCC CTC CAA AAT-3′; GAPDH rev, 5′-GGC TGT TGT CAT ACT TCT CAT GG-3′; CD160 fwd, 5′-GCC AGA AGC CAG AAG TCA GGT ATC CG-3′; CD160 rev, 5′-CCT GTG CCC TGT TGC ATT CTT C-3′; CD58 fwd, 5′-AGA GCA TTA CAA CAG CCA TCG-3′; CD58 rev, 5′-ATC TGT GTC TTG AAT GAC CGC-3′; TNFRSF14 (HVEM) fwd, 5′-GTG CAG TCC AGG TTA TCG TGT-3′; TNFRSF14 rev, 5′-CAC TTG CTT AGG CCA TTG AGG-3′; BTLA fwd, 5′-CAT CTT AGC AGG AGA TCC CTT TG-3′; BTLA rev, 5′-GAC CCA TTG TCA TTA GGA AGC A-3′; TNFSF14 (LIGHT) fwd, 5′-ATA CAA GAG CGA AGG TCT CAC G-3′; TNFSF14 rev, 5′-CTG AGT CTC CCA TAA CAG CGG-3′. Gene expression was evaluated by the comparative CT method and normalized to reference genes.

### Flow Cytometry

Co-signaling molecules usually express high in certain types of immune cells, and their expression levels are intensively related to their immune functions. Because CD160 is mainly expressed in human CD8^+^ T cells, and is weakly expressed in CD4^+^ T cells, B cells and dendritic cells (Supplementary Figures 1, 2), we further studied its expression on the surface of both CD8^+^ T cells and CD4^+^ T cells in GD patients through flow cytometry. To analyze the percentages of CD8^+^CD160^+^ T cells and CD4^+^CD160^+^ T cells, PBMCs were stained with FITC-conjugated anti-CD4 mAb (Becton-Dickinson, 555346), PerCP-Cy5.5-conjugated anti-CD8 mAb (Becton-Dickinson, 565310) and PE-conjugated anti-CD160 mAb (Becton-Dickinson, 562118). PBMCs were incubated at 4°C for 30 min in the dark with the optimal dilution of each antibody according to the manufacturer's instructions. The samples were analyzed on BD FACS Melody™ instrument (Becton-Dickinson).

### Statistical Analysis

Data were shown as mean with standard error (SE) or interquartile range (IQR). Based on the patterns of data distribution, the difference in the expressions of candidate genes or the percentages of immune cells between cases and controls was analyzed using either Mann-Whitney *U-*test or unpaired *t*-test. We further analyzed the diagnostic role of CD160 in autoimmune diseases through receiver operating characteristic (ROC) curve, and the area under the ROC curve (AUCs) were calculated. A two-sided *P* < 0.05 suggested statistically significant difference. Analyses were performed using STATA (version 12.0).

## Results

### Characteristics of Included Datasets

According to the eligibility criteria, a total of 19 array datasets were considered eligible in the RRA discovery study (Table 1). Moreover, 6 RNA-seq datasets were included into the RRA validation study (Table 2). Table 1 showed the main characteristics of those 19 array datasets, such as GEO accession IDs and samples information (Table 1). Table 2 showed the main characteristics of those 6 RNA-seq datasets (Table 2). Those 25 datasets included a total of 2,292 patients with autoimmune diseases and 690 controls. Among those 19 array datasets (Table 1), 7 datasets (GSE121239, GSE65391, GSE61635, GSE80060, GSE93272, GSE17590, and GSE9006) longitudinally profiled the transcriptome of some patients with different visits. To exclude the possible impact of treatment on the expressions of those co-signaling molecules, we only used data of the first visit for those patients. There were no longitudinal gene expression values in those 6 RNA-Seq datasets.

**Table 1:** Characteristics of 19 publicly available array datasets in the RRA discovery study.

**GSE ID**	**Participants**	**Tissues**	**Type of analysis**	**Platform**
GSE121239	65 SLE patients and 20 controls	PBMCs	Array	GPL13158 (Affymetrix)
GSE65391	118 SLE patients and 32 controls	Whole blood	Array	GPL10558 (Illumina)
GSE81622	30 SLE patients and 25 controls	PBMCs	Array	GPL10558 (Illumina)
GSE50772	61 SLE patients and 20 controls	PBMCs	Array	GPL570 (Affymetrix)
GSE61635	79 SLE patients and 30 controls	Whole blood	Array	GPL570 (Affymetrix)
GSE49454	157 SLE patients and 20 controls	Whole blood	Array	GPL10558 (Illumina)
GSE17755SLE	22 SLE cases and 45 controls	Whole blood	Array	GPL1291 (Hitachisoft)
GSE80060	104 JIA patients and 22 controls	Whole blood	Array	GPL570 (Affymetrix)
GSE66795	131 Sjögren's syndrome patients and 29 controls	Whole blood	Array	GPL10558 (Illumina)
GSE84844	30 Sjögren's syndrome patients and 30 controls	Whole blood	Array	GPL570 (Affymetrix)
GSE73754	52 Ankylosing spondylitis patients and 20 controls	Whole blood	Array	GPL10558 (Illumina)
GSE51092	190 Sjögren's syndrome patients and 32 controls	Whole blood	Array	GPL6884 (Illumina)
GSE17755RA	112 RA cases and 45 controls	Whole blood	Array	GPL1291 (Hitachisoft)
GSE45291	493 RA patients and 20 controls	Whole blood	Array	GPL13158 (Affymetrix)
GSE93272	65 RA patients and 35 controls	Whole blood	Array	GPL570 (Affymetrix)
GSE17755JIA	57 JIA cases and 53 controls	Whole blood	Array	GPL1291 (Hitachisoft)
GSE17590	22 JIA cases and 21 controls	Whole blood	Array	GPL6106 (Illumina)
GSE9006	43 T1DM cases and 24 controls	PBMCs	Array	GPL96 (Affymetrix)
GSE3365	85 IBD patients and 42 controls	PBMCs	Array	GPL96 (Affymetrix)

*SLE, systemic lupus erythematosus; RA, rheumatoid arthritis; IBD, inflammatory bowel disease; JIA, juvenile idiopathic arthritis; T1DM, type 1 diabetes mellitus; PBMCs, peripheral blood mononuclear cells; RRA, Robust rank aggregation*.

**Table 2:** Characteristics of 6 RNA-seq datasets in the RRA validation study.

**GSE ID**	**Participants**	**Tissues**	**Type of analysis**	**Platform**
GSE122459	20 SLE patients and 6 controls	PBMCs	RNA-seq	Illumina HiSeq 2500
GSE112087	31 SLE patients and 29 controls	Whole blood	RNA-seq	Illumina HiSeq 2500
GSE117769	50 RA patients and 50 controls	Whole blood	RNA-seq	Illumina HiSeq 2500
GSE112057IBD	75 IBD patients and 12 controls	Whole blood	RNA-seq	Illumina HiSeq 2000
GSE112057JIA	115 JIA patients and 12 controls	Whole blood	RNA-seq	Illumina HiSeq 2000
GSE79970	85 JIA patients and 16 controls	PBMCs	RNA-seq	Illumina HiSeq 2500

*SLE, systemic lupus erythematosus; RA, rheumatoid arthritis; IBD, inflammatory bowel disease; JIA, juvenile idiopathic arthritis; PBMCs, peripheral blood mononuclear cells; RRA, Robust rank aggregation*.

### Outcomes in the RRA Discovery Study

**Figure 1:**
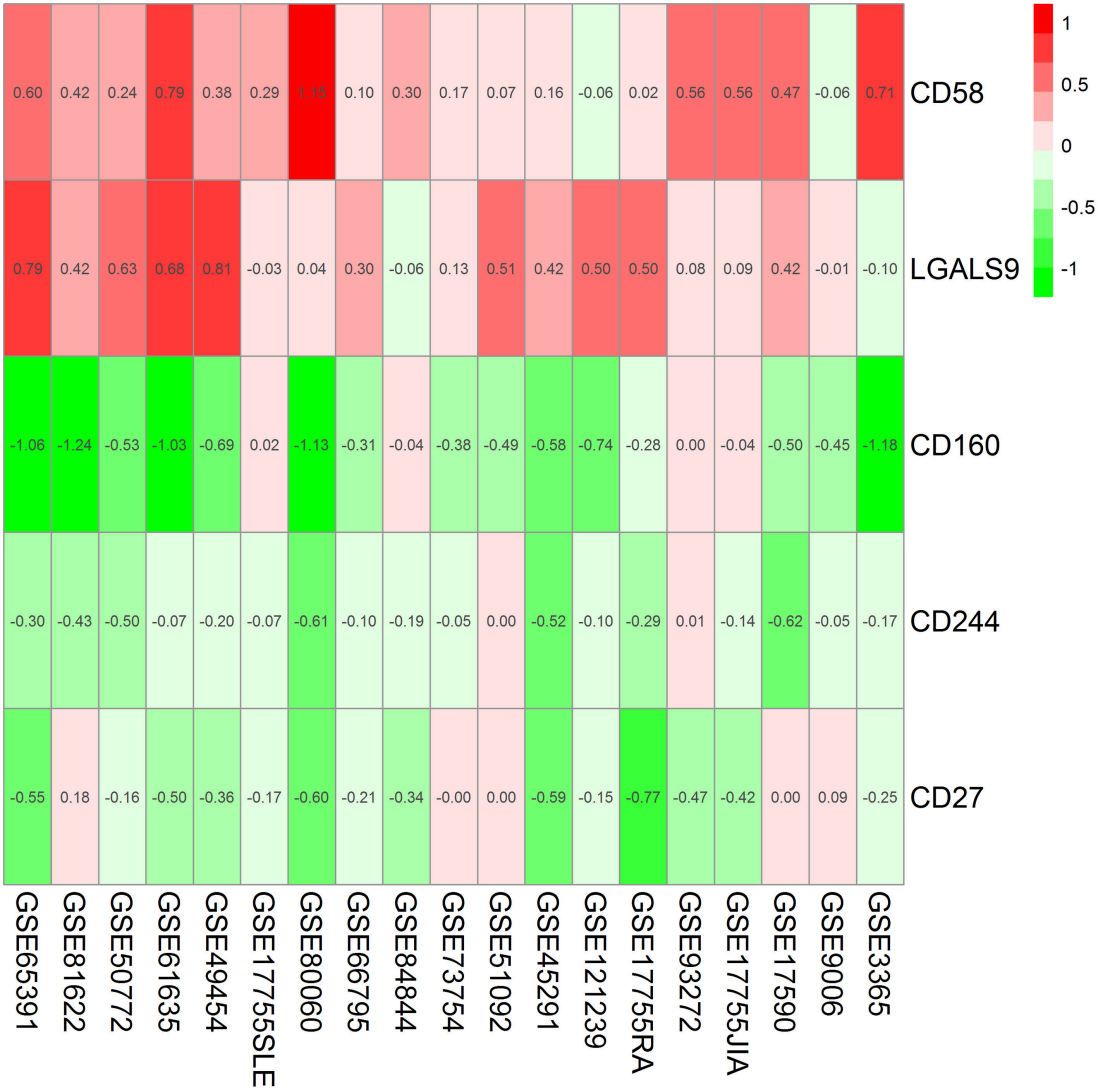
Heatmap shows those significant genes in the RRA analysis of 19 array datasets. Data from 19 array datasets were integrated using RRA method. CD160 was the most significant down-regulated gene in autoimmune diseases (Adjusted *P* = 5.9E-12), while CD58 was the most significant up-regulated gene (Adjusted *P* = 5.7E-06). The numbers in the heatmap were for the logarithmic fold change in each dataset which was calculated by the limma package. Red indicated increased expression, and green indicated decreased expression.

Findings from the RRA discovery study suggested that CD160 was the most significant gene aberrantly expressed in autoimmune diseases (Adjusted *P* = 5.9E-12), followed by CD58 (Adjusted *P* = 5.7E-06), CD244 (Adjusted *P* = 9.5E-05), LGALS9 (Adjusted *P* = 0.001) and CD27 (Adjusted *P* = 0.005) (Figure 1; Table 3). As shown in Figure 1, CD160 was down-regulated in most included datasets (Figure 1). The RRA outcomes after ComBat normalization was similar with that before normalization, and CD160 was still the most aberrantly expressed co-signaling molecule in autoimmune diseases (adjusted *P-*value = 1.3E-11, Supplementary Figure 3, Supplementary Table 4).

**Table 3:** Significant aberrantly expressed genes in the RRA analysis of 19 array datasets.

**Gene name**	**Expression change**	**logFC**	***P-*value**	**adjPvalue**
CD160	Down	−0.56	1.1E-13	5.9E-12
CD58	Up	0.36	1.1E-07	5.7E-06
CD244	Down	−0.23	1.8E-06	9.5E-05
LGALS9	Up	0.32	1.9E-05	0.001
CD27	Down	−0.28	9.1E-05	0.005

*logFC, the log2 of fold change; adjPvalue, adjusted P value; RRA, Robust rank aggregation*.

In the subgroup analyses, both RRA analysis of 14 array datasets using whole blood and RRA analysis of 5 array datasets using PBMCs validated CD160 as the most aberrantly expressed co-signaling molecule in autoimmune diseases (Supplementary Tables 5, 6, Supplementary Figures 4, 5), which suggested that the aberrant expression of CD160 in autoimmune diseases was not affected by the type of transcriptomes. Additionally, both RRA analysis of 9 Affymetrix datasets and RRA analysis of 7 Illumina datasets consistently identified CD160 as the most aberrantly expressed co-signaling molecule in autoimmune diseases (Supplementary Tables 7, 8, Supplementary Figures 6, 7), which suggested that the aberrant expression of CD160 in autoimmune diseases was also not significantly influenced by types of platforms (Illumina or Affymetrix).

### Outcomes in the RRA Validation Study

**Figure 2:**
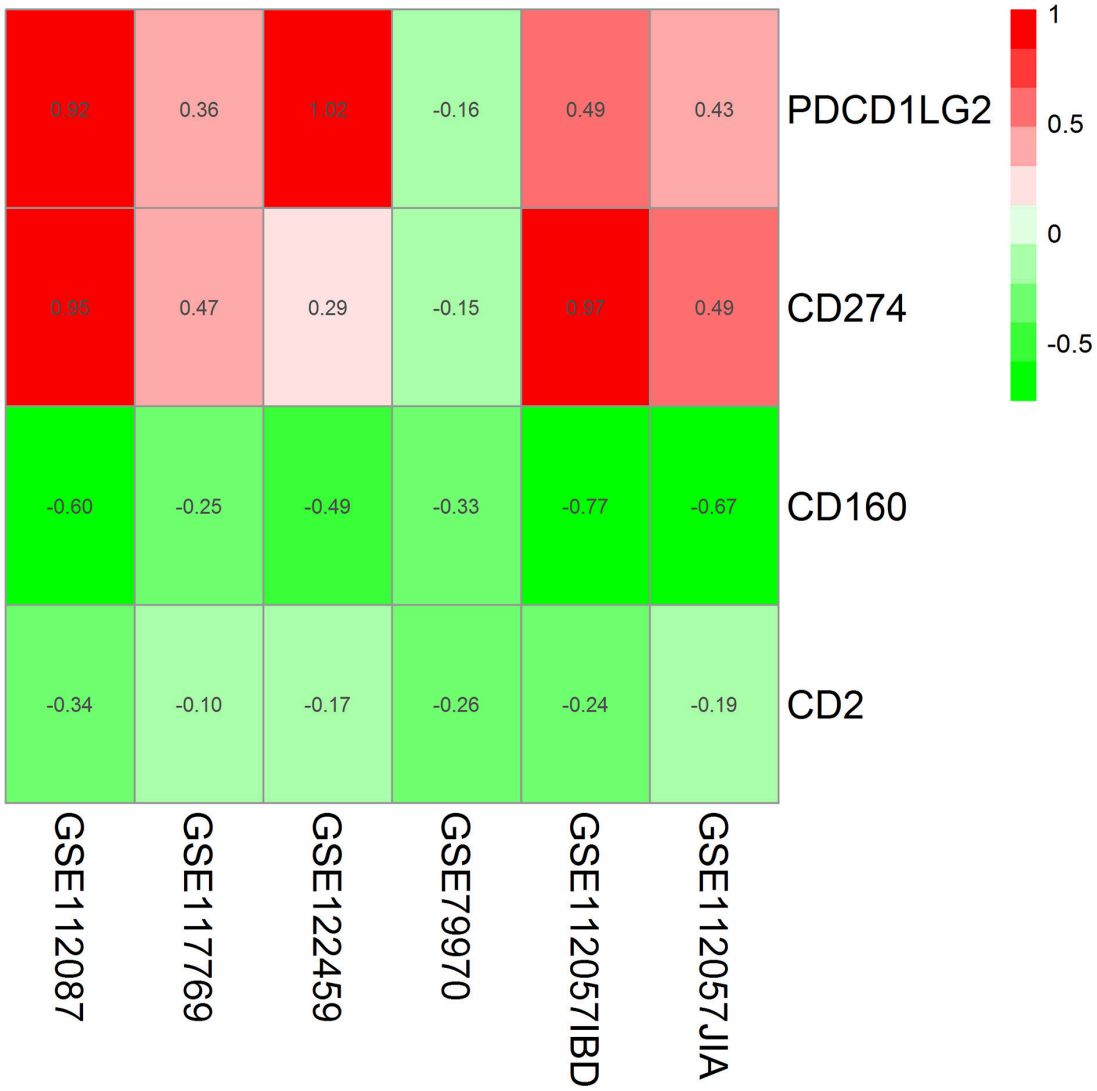
Heatmap shows those significant genes in the RRA analysis of 6 RNA-seq datasets. Data from 6 RNA-seq datasets were integrated using RRA method. CD160 was the most significant down-regulated gene in autoimmune diseases (Adjusted *P* = 5.9E-09), while CD58 was not an obviously significant gene in the RRA validation study. The numbers in the heatmap were for the logarithmic fold change in each dataset which was calculated by the limma package. Red indicated increased expression, and green indicated decreased expression.

In the RRA validation study, RRA analysis of those 6 RNA-seq datasets validated CD160 as the most significant gene aberrantly expressed in autoimmune diseases (Adjusted *P* = 5.9E-09) (Figure 2, Table 4). However, findings from the RRA validation study suggested that CD58 was not aberrantly expressed in autoimmune diseases (Adjusted *P* = 0.99).

**Table 4:** Significant aberrantly expressed genes in the RRA analysis of 6 RNA-seq datasets.

**Gene name**	**Expression change**	**logFC**	***P-*value**	**adjPvalue**
CD160	Down	−0.52	1.1E-06	5.9E-09
PDCD1LG2	Up	0.51	0.0006	0.03
CD274	Down	0.50	0.0008	0.04

*logFC, the log2 of fold change; adjPvalue, adjusted P value; RRA, Robust rank aggregation*.

### Validation in SLE, RA, IBD, and JIA

**Figure 3:**
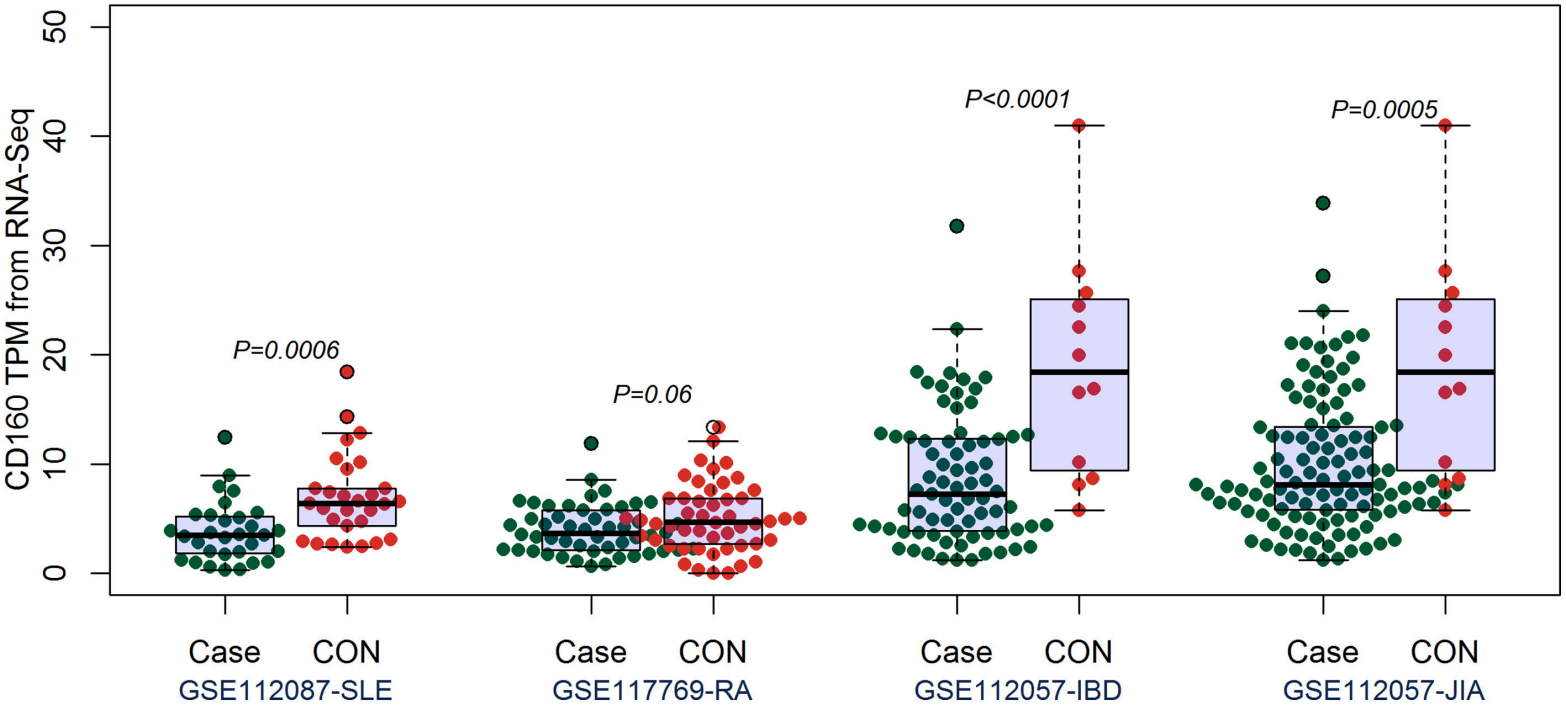
Validation of the aberrant expression of CD160 in SLE, RA, IBD, and JIA patients. The transcripts per million (TPM) value of CD160 were calculated from the raw read counts of four RNA-seq datasets including SLE (GSE112087), RA (GSE117769), IBD (GSE112057IBD), and JIA (GSE112057JIA), respectively. Difference between groups was analyzed using unpaired *t-*test. The aberrant expression of CD160 was statistically significant in SLE (*P* = 0.0006), IBD (*P* < 0.0001), and JIA (*P* = 0.0005), and was marginally significant in RA (*P* = 0.06).

**Figure 4:**
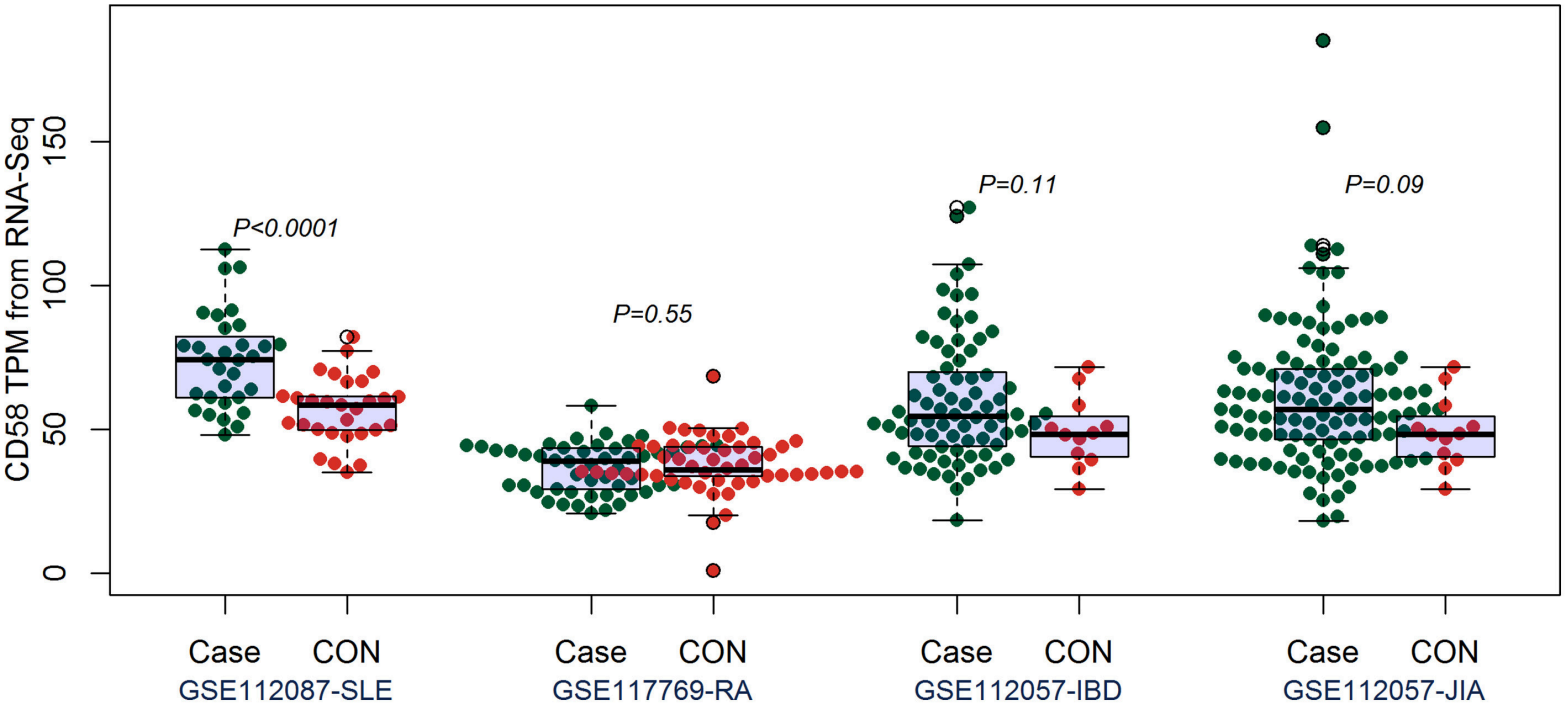
Validation of the expression of CD58 in SLE, RA, IBD, and JIA patients. The transcripts per million (TPM) value of CD58 were calculated from the raw read counts of four RNA-seq datasets including SLE (GSE112087), RA (GSE117769), IBD (GSE112057IBD), and JIA (GSE112057JIA), respectively. Difference between groups was analyzed using unpaired *t-*test. CD58 was aberrantly expressed between SLE patients and controls (*P* < 0.0001), but it not obvious in RA (*P* = 0.55), IBD (*P* = 0.11), and JIA (*P* = 0.09).

By comparing the TPM values of CD160 and CD58 from four RNA-seq datasets including SLE (GSE112087), RA (GSE117769), IBD (GSE112057IBD), and JIA (GSE112057JIA), we found that the aberrant expression of CD160 was statistically significant in SLE (*P* = 0.0006), IBD (*P* < 0.0001) and JIA (*P* = 0.0005), and was marginally significant in RA (*P* = 0.06) (Figure 3). CD58 was aberrantly expressed between SLE patients and controls (*P* < 0.0001), but it not obvious in RA (*P* = 0.55), IBD (*P* = 0.11), and JIA (*P* = 0.09) (Figure 4).

**Figure 5:**
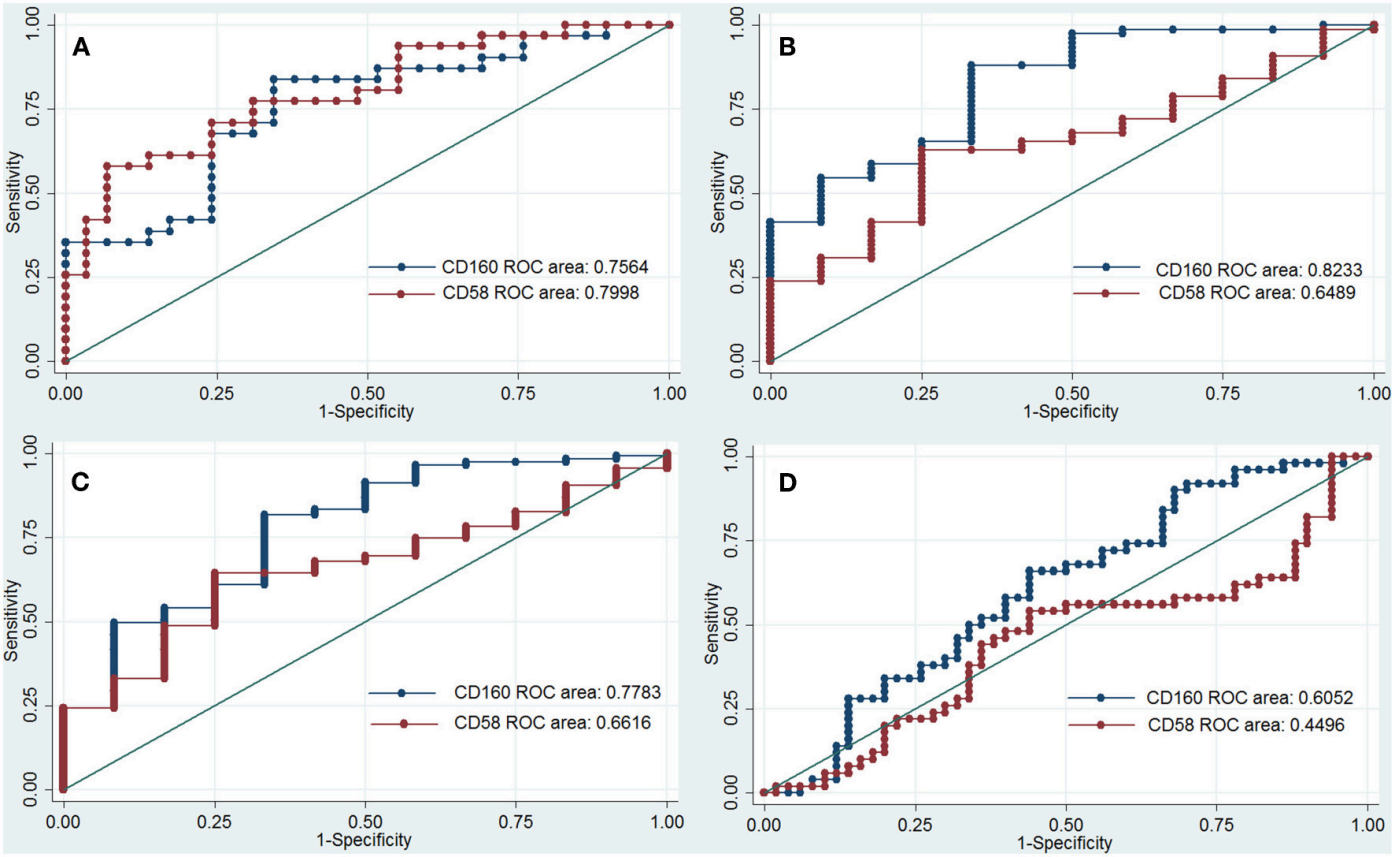
ROC curve to assess the diagnostic roles of CD160 and CD58 in autoimmune diseases. The diagnostic roles of CD160 and CD58 in autoimmune diseases were assessed by receiver operating characteristic (ROC) curve. Transcripts per million (TPM) values of CD58 and CD160 in four RNA-seq datasets including SLE (GSE112087), RA (GSE117769), IBD (GSE112057IBD), and JIA (GSE112057JIA) were used. The area under the ROC curve (AUCs) were shown in the figures. **(A)** Shows the diagnostic roles of CD160 and CD58 in SLE, and the AUC for CD160 and CD58 in diagnosing SLE is 0.756 (95%CI 0.63–0.88) and 0.799 (95%CI 0.69–0.91), respectively. When the testing threshold for the TPM value of CD160 was defined as 4.35, the sensitivity and specificity for CD160 in diagnosing RA were 75.9 and 67.7%, respectively. **(B)** Shows the diagnostic roles of CD160 and CD58 in IBD, and the AUC for CD160 and CD58 in diagnosing IBD is 0.823 (95%CI 0.69–0.95) and 0.649 (95%CI 0.50–0.80), respectively. When the testing threshold for the TPM value of CD160 was defined as 8.67, the sensitivity and specificity for CD160 in diagnosing IBD were 83.3 and 58.7%, respectively. **(C)** Shows the diagnostic roles of CD160 and CD58 in JIA, and the AUC for CD160 and CD58 in diagnosing JIA is 0.778 (95%CI 0.63–0.92) and 0.662 (95%CI 0.53–0.80), respectively. When the testing threshold for the TPM value of CD160 was defined as 8.67, the sensitivity and specificity for CD160 in diagnosing JIA were 83.3 and 53.9%, respectively. **(D)** Shows the diagnostic roles of CD160 and CD58 in RA, and the AUC for CD160 and CD58 in diagnosing RA is 0.605 (95%CI 0.49–0.72) and 0.449 (95%CI 0.33–0.57), respectively.

CD160 had a moderate role in diagnosing SLE, IBD and JIA, and the AUCs for CD160 to diagnose SLE, IBD, and JIA were 0.756 (95%CI 0.63–0.88), 0.823 (95%CI 0.69–0.95) and 0.778 (95%CI 0.63–0.92) (Figure 5), respectively. CD58 had a moderate role in diagnosing SLE with an AUC of 0.799 (95%CI 0.69–0.91), but it had poor value in diagnosing RA, JIA, and IBD (Figure 5).

### Aberrant Expression of CD160 in GD

**Figure 6:**
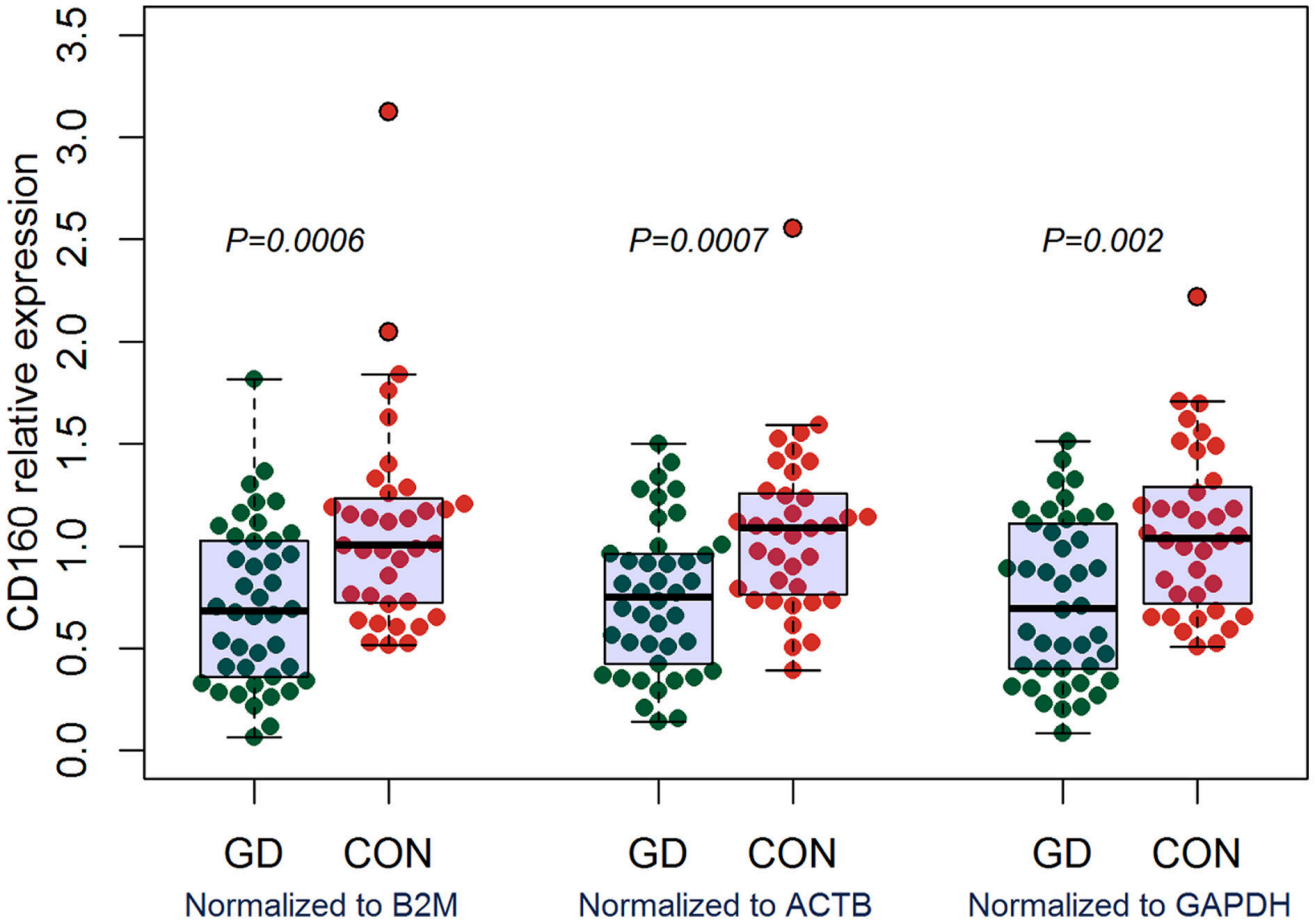
qRT-PCR outcomes suggested that CD160 was obviously aberrantly expressed in GD patients. Expression level of CD160 in the PBMCs were determined using qRT-PCR. Gene expression levels were calculated using the comparative CT method and were normalized to three reference genes including B2M, ACTB, and GAPDH, respectively. There were a total of 42 GD patients and 36 healthy controls. Difference between groups was analyzed using Mann-Whitney *U-*test.

**Figure 7:**
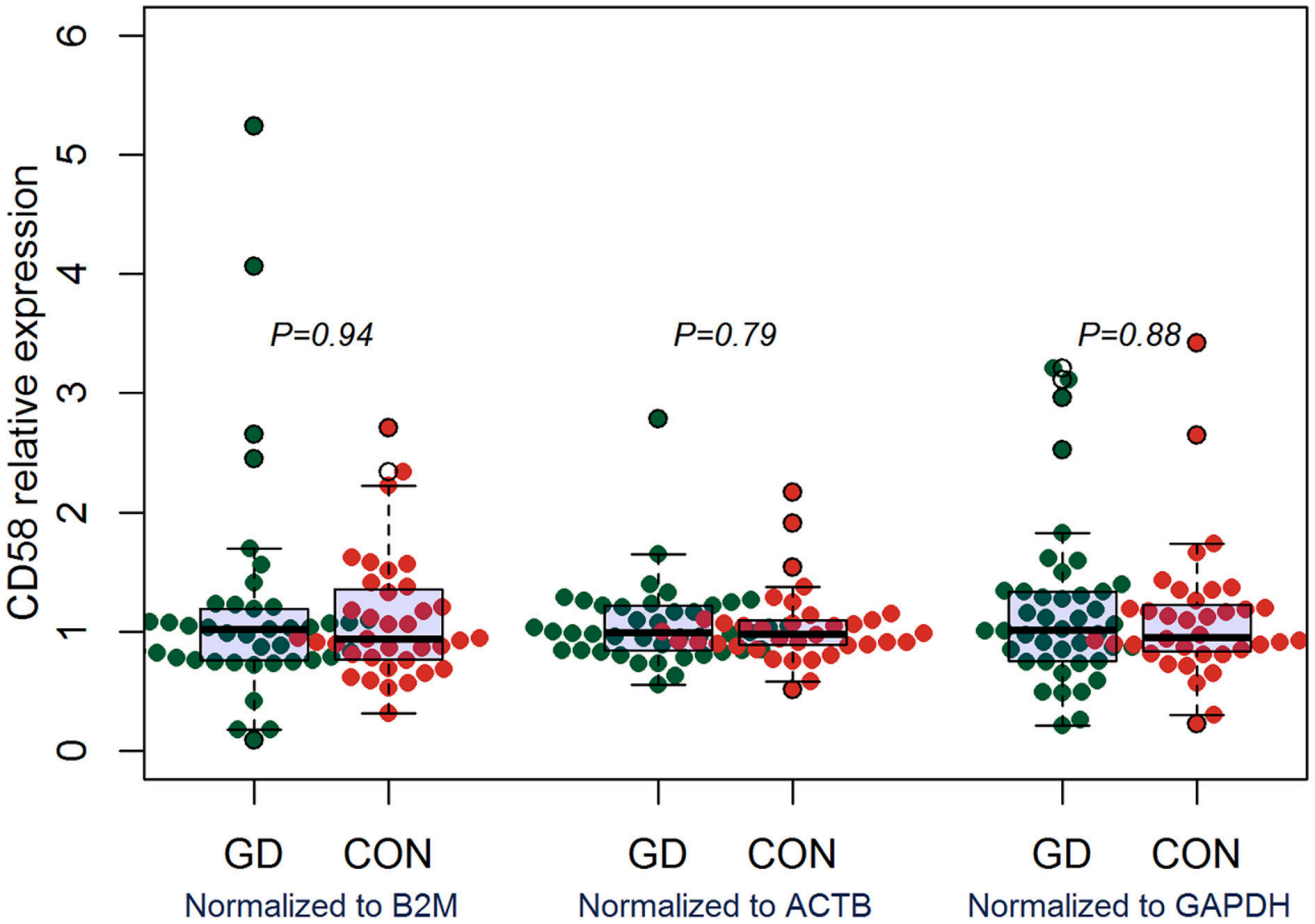
qRT-PCR outcomes suggested that CD58 was not aberrantly expressed in GD patients. Expression level of CD58 in the PBMCs were determined using qRT-PCR. Gene expression levels were calculated using the comparative CT method and were normalized to three reference genes including B2M, ACTB, and GAPDH, respectively. There were a total of 42 GD patients and 36 healthy controls. Difference between groups was analyzed using Mann-Whitney *U-*test.

**Figure 8:**
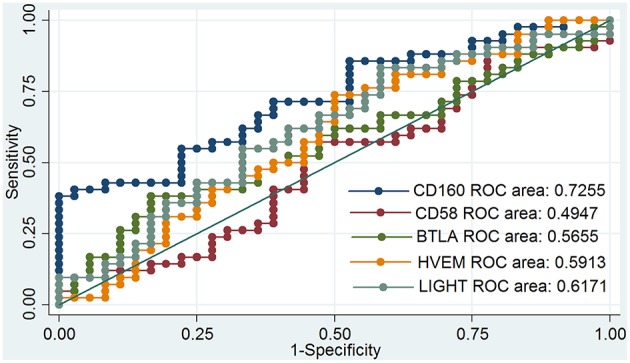
ROC curve to assess the diagnostic roles of CD160, CD58, BTLA, HVEM, and LIGHT in GD. The diagnostic roles of CD160, CD58, BTLA, HVEM, and LIGHT in GD were assessed by receiver operating characteristic (ROC) curve. There were a total of 42 GD patients and 36 healthy controls. The area under the ROC curve (AUCs) were shown in the figure, and CD160 had the best diagnostic role among those 5 genes with an AUC of 0.725. When the testing threshold was defined as 0.5 for CD160, the sensitivity and specificity for CD160 in diagnosing GD were 100.0 and 38.1%, respectively. When the testing threshold was defined as 1.0 for CD160, the sensitivity and specificity for CD160 in diagnosing GD were 50.0 and 71.4%, respectively.

**Figure 9:**
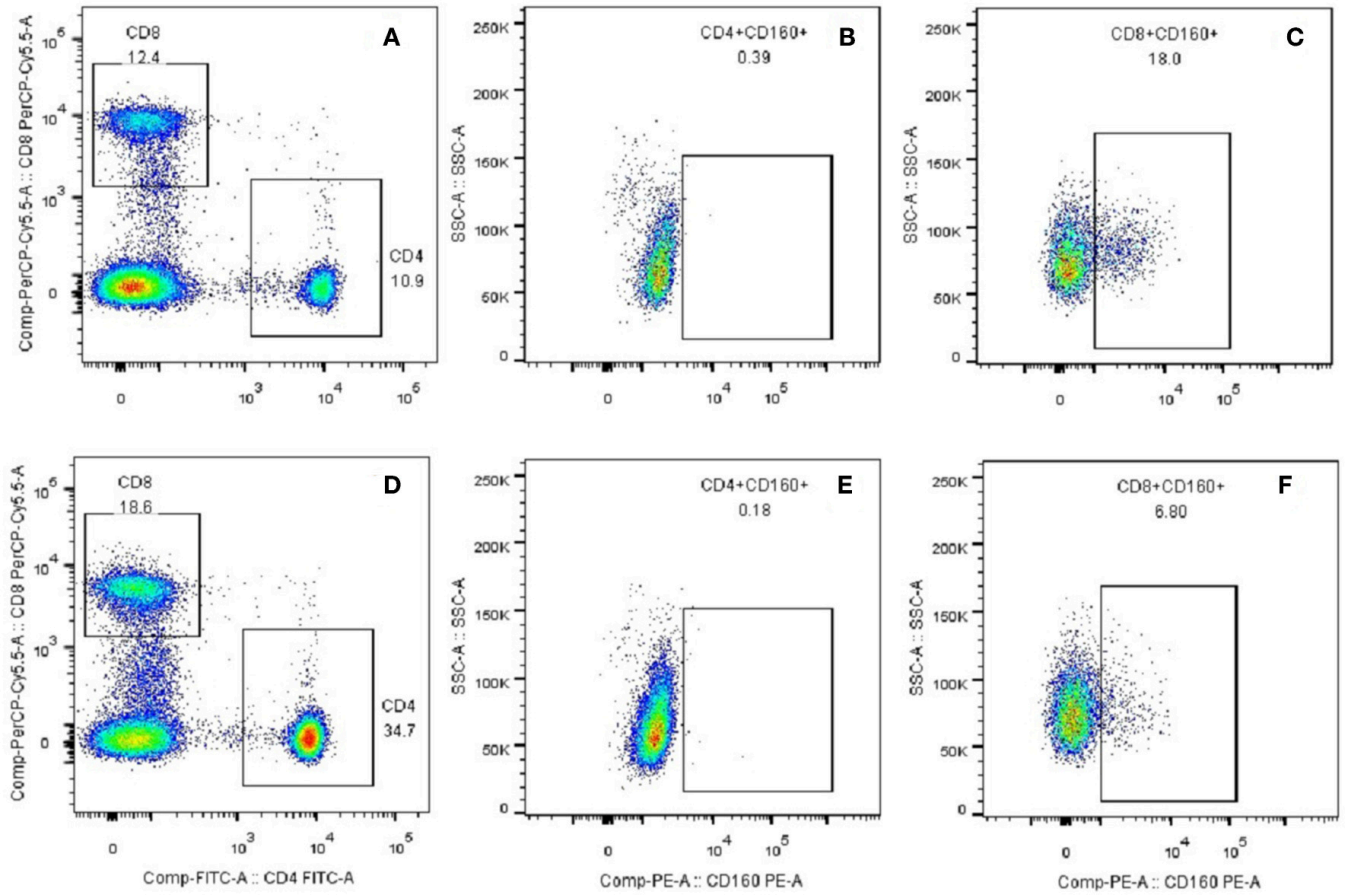
Plots of CD8^+^CD160^+^ T cells and CD4^+^CD160^+^ T cells from flow cytometry. **(A)** CD4^+^ and CD8^+^ T subsets were gated by flow cytometry in the PBMCs of healthy controls. **(B)** CD4^+^CD160^+^ T cells were gated by flow cytometry in the PBMCs of healthy controls. **(C)** CD8^+^CD160^+^ T cells were gated by flow cytometry in the PBMCs of healthy controls. **(D)** CD4^+^ and CD8^+^ T subsets were gated by flow cytometry in the PBMCs of GD patients. **(E)** CD4^+^CD160^+^ T cells were gated by flow cytometry in the PBMCs of GD patients. **(F)** CD8^+^CD160^+^ T cells were gated by flow cytometry in the PBMCs of GD patients.

**Figure 10:**
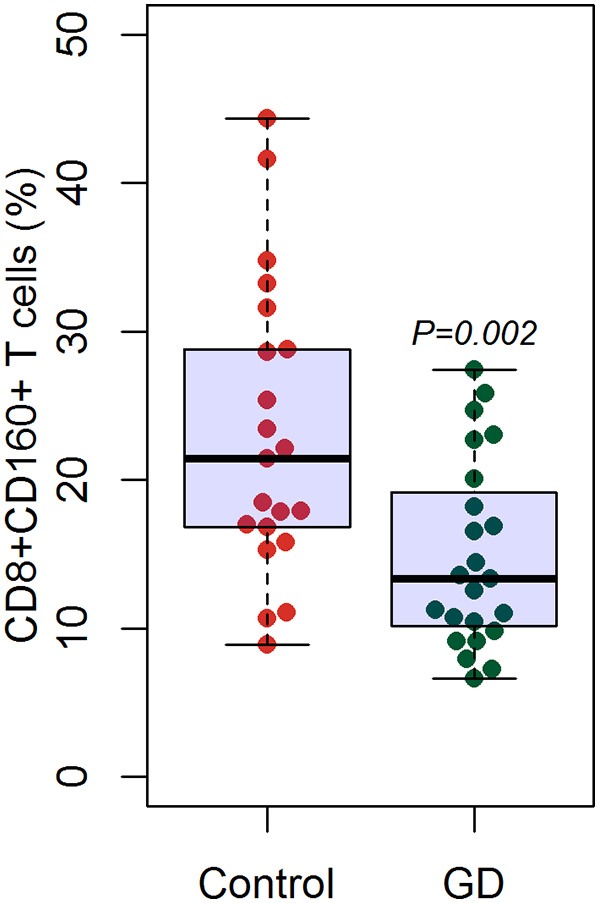
Flow cytometry suggested decreased percentage of CD8^+^CD160^+^ T cells in GD patients than healthy controls. There were a total of 23 GD patients and 21 healthy controls. Difference between groups was analyzed using unpaired *t*-test.

Outcomes of qRT-PCR suggested that CD160 was aberrantly expressed in the PBMCs between GD patients and healthy controls (*P* < 0.05), but CD58, BTLA, LIGHT, and HVEM were not (*P* > 0.05) (Figures 6, 7, Supplementary Figure 8). Moreover, CD160 had a moderate role in diagnosing GD, and the AUC was 0.725 (95%CI 0.61 to 0.84) (Figure 8). When the testing threshold was defined as 0.5 for CD160, the sensitivity and specificity for CD160 in diagnosing GD were 100.0 and 38.1%, respectively. When the testing threshold was defined as 1.0 for CD160, the sensitivity and specificity for CD160 in diagnosing GD were 50.0 and 71.4%, respectively. The outcomes from flow cytometry confirmed that CD160 was differentially expressed on the surface of CD8^+^ T cells between GD patients and healthy controls and the percentage of CD8^+^CD160^+^ T cells was obviously lower in GD patients than that of healthy controls (*P* = 0.002, Figures 9, 10), which proved the aberrant expression of CD160 in GD at the protein level.

## Discussion

Co-signaling molecules play essential roles in modulating T cells-mediated immune responses, but their roles in autoimmune diseases has not yet clearly defined. We systematically assessed the expressions of 54 co-stimulatory or co-inhibitory genes in 2,292 patients with autoimmune diseases and 690 controls. The study suggests that CD160 is the most significant co-signaling gene aberrantly expressed in autoimmune diseases, and the aberrant expression of CD160 is an important characteristic of autoimmunity including GD, which indicates that the dysfunction of CD160-related pathway exerts a critical role in the development of autoimmunity.

Excessive co-stimulation and/or insufficient co-inhibitory signaling can result in the activation of autoimmune cells and thus lead to the onset of autoimmunity. Previous studies have suggested that some autoimmune diseases are caused by the disruption of the balance between co-stimulation and co-inhibition signaling pathways (21). Some studies showed that some co-stimulatory or co-inhibitory genes were aberrantly expressed in patients with autoimmune diseases, such as Programmed Death-Ligand 1 (PDL1), Programmed cell death-1 (PD-1), and CD40 (39–42). For instance, PD1 pathway was down-regulated in several autoimmune diseases such as RA, multiple sclerosis and T1DM (43–45), while inducible T cell costimulator (ICOS) is highly expressed in patients with SLE, IBD, or RA (46–48). Moreover, *in vivo* studies also have proven knock-out or blockade of some co-stimulatory or co-inhibitory genes can promote or delay the development of autoimmunity, such as Cytotoxic T-Lymphocye Antigen 4 (CTLA-4), CD40 ligand (CD40L), and CD28 (49–53). Finally, findings from gene-association studies also have suggested that some polymorphisms in co-stimulatory or co-inhibitory genes are intensively related to risk of autoimmune diseases, such as *CLTA4, CD40*, and *CD28* (54–58). Therefore, dysfunction of co-stimulatory and co-inhibitory pathways exerts critical roles in the pathogeneses of autoimmunity.

The findings in the present study suggest the critical role of CD160 in the pathogenesis of autoimmunity. CD160 is a member of an emerging co-stimulatory/co-inhibitory pathway, namely HVEM/CD160/BTLA/LIGHT pathway (59–64). HVEM is known as tumor necrosis factor receptor superfamily member 14 (TNFRSF14) and has dual functional activity by either binding to co-inhibitory receptors (CD160 and BTLA), or binding to a co-stimulatory receptor LIGHT (65). The combination of LIGHT with HVEM exhibits co-stimulatory signal and can promote the immune response, while the binding of BTLA or CD160 to HVEM exhibits co-inhibitory signal and can attenuate TCR signals (66). Therefore, HVEM/ CD160/BTLA/LIGHT pathway is a bidirectional switch which can critically regulate the activation of T cells (66). Some studies have indicated HVEM/CD160/BTLA/LIGHT pathway may has an important role in the etiology of autoimmunity. For instance, several studies using animal models of autoimmune diseases proved the involvement of HVEM/CD160/BTLA/LIGHT pathway in the development of autoimmune diseases (67–71). Additionally, the aberrantly expression of HVEM, BTLA, and LIGHT has been studied in some autoimmune diseases, such as RA, T1DM, and SLE, and there was disease-specific difference in the outcomes (63, 72–75). Additionally, this study also suggest that CD160 has a moderate role in diagnosing SLE, IBD, JIA, and GD (Figures 5, 8), suggesting that CD160 may be helpful for the diagnosis of autoimmune diseases. Most autoimmune diseases are difficult to diagnose, and there is still lack of good biomarkers for many autoimmune diseases. The AUC of the ROC analysis for CD160 was generally less than 0.80, suggesting that it only had a moderate role in diagnosing autoimmune diseases and should not be used in a clinical setting as a sole diagnostic marker. The value of the combination of CD160 with previously established diagnostic methods in the diagnosis of common autoimmune diseases may be promising, and can be explored in future studies.

There are few studies focusing on the role of CD160 in autoimmune diseases. A study by Hosokawa et al. reported that CD160 was aberrantly expressed in CD8^+^ memory stem T cells (TSCMs) of acquired aplastic anemia (AA) patients (76). Hosokawa et al. also reported that the percentage of CD8^+^CD160^+^ TSCMs was not different between SLE patients and controls (*P* > 0.05) (76), but the percentage of CD8^+^CD160^+^ T cells was not analyzed. A gene-association study suggested that CD160 rs744877 was associated with RA, suggesting the involvement of CD160 in the pathogenesis of RA (77). Another study by Bouma et al. revealed that Thiopurine treatment reduced the expression of CD160 in whole blood of Crohn's disease patients, and thiopurine might produce its effect by selectively affecting effector cytotoxic CD160-positive cells (78). Collectively, the role of CD160 in the pathogenesis of autoimmunity is still largely rudimentary. Besides, few studies have investigated the expression of CD160 in those autoimmune diseases using qRT-PCR or flow cytometry, and additional studies are needed.

Some studies suggested that several co-stimulatory/co-inhibitory molecules were involved in the pathogenesis of GD, such as CTLA4, CD40, ICOS, and ICOSL (79–83). Our study firstly assessed the expression levels of HVEM, CD160, BTLA, and LIGHT in GD patients, and found that CD160 was aberrantly expressed in GD patients (Figure 6), which had not been reported in previous studies. The outcomes from flow cytometry confirmed that the percentage of CD8^+^CD160^+^ T cells was obviously lower in GD patients than that of healthy controls (Figure 10), which proved the aberrant expression of CD160 in GD at the protein level. Our findings also suggested that CD160 had a moderate role in diagnosing GD. However, the clinical significance of CD160 in GD is still not well defined, and further studies are needed.

A better understanding of the roles of co-stimulatory/co-inhibitory pathways in autoimmune diseases have important implications for the development of novel therapeutics (84, 85). Currently, some therapeutic interventions by targeting aberrantly expressed co-stimulatory/co-inhibitory genes have been studied as promising therapeutic methods for autoimmune diseases, such anti-CD40L drugs and CTLA-4Ig (86–89). The findings from our study identify CD160 as a novel therapeutic target for the treatment of autoimmune diseases, which has important implications for future studies on CD160-related pathway. Future studies are recommended to explore the feasibility of treating autoimmune diseases by targeting CD160-related pathway.

In our study, we found that CD160 was the most significant co-signaling gene aberrantly expressed in autoimmune diseases, while several widely-studied co-signaling genes, such as ICOS, PD1 and CLTA4, were not identified as significant genes in the RRA analysis. One possible explanation is the disease-specific roles of those co-signaling genes in autoimmune diseases. For instance, PD1 was differentially expressed on the CD4^+^ and CD8^+^ T cells between RA and psoriatic arthritis patients (90). Another possible explanation is the use of transcriptome data in whole blood or PBMC but not in specific types of immune cells. There is high possibility for the existence of aberrant expression of some co-signaling genes in certain types of immune cells but not in whole blood or PBMCs. Owing to the limited transcriptome datasets from specific types of immune cells in GEO, we were unable to analyze the differentially expressed genes in specific types of immune cells by RAA. Therefore, further studies are recommended to explore autoimmunity-related co-signaling genes in specific types of immune cells for the existence of enough transcriptome datasets in the future. Besides, the results for other molecules such as CD58 and CD244 were not consistent in our subgroup analyses by types of datasets, which may be resulted from the moderate difference between cases and controls or the difference in the compositions of immune cells between whole blood and PBMCs. The expression levels of these co-signaling molecules in autoimmune diseases need to be explored in more future studies.

This study suggests that CD160 is the most significant co-signaling gene aberrantly expressed in autoimmune diseases, and its dysfunction is an important characteristic of autoimmunity including GD. However, the molecular mechanism underlying the role of CD160 in autoimmunity is largely elusive, and additional studies are warranted to uncover it. Moreover, future studies are recommended to explore the feasibility of treating autoimmune diseases by targeting CD160-related pathway.

## Author Contributions

WH, JZ, and SL designed the study, collected data, performed statistical analyses, and wrote the final version of the manuscript. BW and QL participated in data collection and performed statistical analyses. XJ, QY, and RS participated in data collection. All authors approved the final version of the manuscript.

### Conflict of Interest Statement

The authors declare that the research was conducted in the absence of any commercial or financial relationships that could be construed as a potential conflict of interest.

## References

[1] Ji J Sundquist J Sundquist K. Gender-specific incidence of autoimmune diseases from national registers. J Autoimmun. (2016) 69:102–6. 10.1016/j.jaut.2016.03.003 26994904

[2] Ramos-Casals M Brito-Zeron P Kostov B Siso-Almirall A Bosch X Buss D. Google-driven search for big data in autoimmune geoepidemiology: analysis of 394,827 patients with systemic autoimmune diseases. Autoimmun Rev. (2015) 14:670–9. 10.1016/j.autrev.2015.03.008 25842074

[3] Di Sabatino A Lenti MV Giuffrida P Vanoli A Corazza GR New insights into immune mechanisms underlying autoimmune diseases of the gastrointestinal tract. Autoimmun Rev. (2015) 14:1161–9. 10.1016/j.autrev.2015.08.004 26275585

[4] Lisnevskaia L Murphy G Isenberg D. Systemic lupus erythematosus. Lancet (2014) 384:1878–88. 10.1016/S0140-6736(14)60128-8 24881804

[5] Mastrandrea LD. An overview of organ-specific autoimmune diseases including immunotherapy. Immunol Invest. (2015) 44:803–16. 10.3109/08820139.2015.1099409 26575465

[6] Dahan S Segal Y Shoenfeld Y. Dietary factors in rheumatic autoimmune diseases: a recipe for therapy? Nat Rev Rheumatol. (2017) 13:348–58. 10.1038/nrrheum.2017.42 28405001

[7] Gutierrez-Arcelus M Rich SS Raychaudhuri S. Autoimmune diseases - connecting risk alleles with molecular traits of the immune system. Nat Rev Genet. (2016) 17:160–74. 10.1038/nrg.2015.33 26907721 PMC4896831

[8] Inshaw JRJ Cutler AJ Burren OS Stefana MI Todd JA. Approaches and advances in the genetic causes of autoimmune disease and their implications. Nat Immunol. (2018) 19:674–84. 10.1038/s41590-018-0129-8 29925982

[9] Stagi S Rigante D. Vitamin D and juvenile systemic lupus erythematosus: Lights, shadows and still unresolved issues. Autoimmun Rev. (2018) 17:290–300. 10.1016/j.autrev.2018.01.004 29353100

[10] Nielsen PR Kragstrup TW Deleuran BW Benros ME. Infections as risk factor for autoimmune diseases - A nationwide study. J Autoimmun. (2016) 74:176–81. 10.1016/j.jaut.2016.05.013 27267460

[11] Jean-Baptiste VSE Xia CQ Clare-Salzler MJ Horwitz MS. Type 1 diabetes and type 1 interferonopathies: localization of a type 1 common thread of virus infection in the pancreas. EBioMedicine (2017) 22:10–7. 10.1016/j.ebiom.2017.06.014 28663145 PMC5552106

[12] Carnero-Montoro E Alarcon-Riquelme ME. Epigenome-wide association studies for systemic autoimmune diseases: the road behind and the road ahead. Clin Immunol. (2018) 196:21–33. 10.1016/j.clim.2018.03.014 29605707

[13] Wang B Shao X Song R Xu D Zhang JA. The emerging role of epigenetics in autoimmune thyroid diseases. Front Immunol. (2017) 8:396. 10.3389/fimmu.2017.00396 28439272 PMC5383710

[14] Kasler HG Lee IS Lim HW Verdin E. Histone Deacetylase 7 mediates tissue-specific autoimmunity via control of innate effector function in invariant natural killer T cells. Elife (2018) 7:e32109. 10.7554/eLife.32109 29664401 PMC5943034

[15] Maldini CR Ellis GI Riley JL. CAR T cells for infection, autoimmunity and allotransplantation. Nat Rev Immunol. (2018) 18:605–16. 10.1038/s41577-018-0042-2 30046149 PMC6505691

[16] Musaelyan A Lapin S Nazarov V Tkachenko O Gilburd B Mazing A. Vimentin as antigenic target in autoimmunity: a comprehensive review. Autoimmun Rev. (2018) 17:926–34. 10.1016/j.autrev.2018.04.004 30009963

[17] Petersone L Edner NM Ovcinnikovs V Heuts F Ross EM Ntavli E. T Cell/B cell collaboration and autoimmunity: an intimate relationship. Front Immunol. (2018) 9:1941. 10.3389/fimmu.2018.01941 30210496 PMC6119692

[18] Prendergast CT Patakas A Al-Khabouri S McIntyre CL McInnes IB Brewer JM. Visualising the interaction of CD4 T cells and DCs in the evolution of inflammatory arthritis. Ann Rheum Dis. (2018) 77:579–88. 10.1136/annrheumdis-2017-212279 29358281

[19] von Burg N Turchinovich G Finke D Maintenance of immune homeostasis through ILC/T cell interactions. Front Immunol. (2015) 6:416 10.3389/fimmu.2015.00416 26322047 PMC4534831

[20] Kumar P Bhattacharya P Prabhakar BS. A comprehensive review on the role of co-signaling receptors and Treg homeostasis in autoimmunity and tumor immunity. J Autoimmun. (2018) 95:77–99. 10.1016/j.jaut.2018.08.007 30174217 PMC6289740

[21] Zhang Q Vignali DA. Co-stimulatory and co-inhibitory pathways in autoimmunity. Immunity (2016) 44:1034–51. 10.1016/j.immuni.2016.04.017 27192568 PMC4873959

[22] Ford ML. T cell cosignaling molecules in transplantation. Immunity (2016) 44:1020–33. 10.1016/j.immuni.2016.04.012 27192567 PMC5260013

[23] Ford ML Adams AB Pearson TC. Targeting co-stimulatory pathways: transplantation and autoimmunity. Nat Rev Nephrol. (2014) 10:14–24. 10.1038/nrneph.2013.183 24100403 PMC4365450

[24] Patsoukis N Weaver JD Strauss L Herbel C Seth P Boussiotis VA. Immunometabolic regulations mediated by coinhibitory receptors and their impact on T cell immune responses. Front Immunol. (2017) 8:330. 10.3389/fimmu.2017.00330 28443090 PMC5387055

[25] Dominguez-Villar M Hafler DA. Regulatory T cells in autoimmune disease. Nat Immunol. (2018) 19:665–73. 10.1038/s41590-018-0120-4 29925983 PMC7882196

[26] Liebmann M Hucke S Koch K Eschborn M Ghelman J Chasan AI. Nur77 serves as a molecular brake of the metabolic switch during T cell activation to restrict autoimmunity. Proc Natl Acad Sci USA. (2018) 115:E8017–26. 10.1073/pnas.1721049115 30072431 PMC6112725

[27] Ansari AW Khan MA Schmidt RE Broering DC. Harnessing the immunotherapeutic potential of T-lymphocyte co-signaling molecules in transplantation. Immunol Lett. (2017) 183:8–16. 10.1016/j.imlet.2017.01.008 28119073

[28] Kolde R Laur S Adler P Vilo J. Robust rank aggregation for gene list integration and meta-analysis. Bioinformatics (2012) 28:573–80. 10.1093/bioinformatics/btr709 22247279 PMC3278763

[29] Yan S Wang W Gao G Cheng M Wang X Wang Z. Key genes and functional coexpression modules involved in the pathogenesis of systemic lupus erythematosus. J Cell Physiol. (2018) 233:8815–25. 10.1002/jcp.26795 29806703

[30] Leek JT Johnson WE Parker HS Jaffe AE Storey JD. The sva package for removing batch effects and other unwanted variation in high-throughput experiments. Bioinformatics (2012) 28:882–3. 10.1093/bioinformatics/bts034 22257669 PMC3307112

[31] Peters TJ French HJ Bradford ST Pidsley R Stirzaker C Varinli H. Evaluation of cross-platform and interlaboratory concordance via consensus modelling of genomic measurements. Bioinformatics (2018). 10.1093/bioinformatics/bty675. [Epub ahead of print]. 30084929 PMC6378945

[32] Zhang W Yu Y Hertwig F Thierry-Mieg J Zhang W Thierry-Mieg D. Comparison of RNA-seq and microarray-based models for clinical endpoint prediction. Genome Biol. (2015) 16:133. 10.1186/s13059-015-0694-1 26109056 PMC4506430

[33] Farkas MH Au ED Sousa ME Pierce EA. RNA-Seq: improving our understanding of retinal biology and disease. Cold Spring Harb Perspect Med. (2015) 5:a017152. 10.1101/cshperspect.a017152 25722474 PMC4561396

[34] Marioni JC Mason CE Mane SM Stephens M Gilad Y. RNA-seq: an assessment of technical reproducibility and comparison with gene expression arrays. Genome Res. (2008) 18:1509–17. 10.1101/gr.079558.108 18550803 PMC2527709

[35] Dillies MA Rau A Aubert J Hennequet-Antier C Jeanmougin M Servant N. A comprehensive evaluation of normalization methods for Illumina high-throughput RNA sequencing data analysis. Brief Bioinform. (2013) 14:671–83. 10.1093/bib/bbs046 22988256

[36] Conesa A Madrigal P Tarazona S Gomez-Cabrero D Cervera A McPherson A A survey of best practices for RNA-seq data analysis. Genome Biol. (2016) 17:13 10.1186/s13059-016-0881-8 26813401 PMC4728800

[37] Wagner GP Kin K Lynch VJ. Measurement of mRNA abundance using RNA-seq data: RPKM measure is inconsistent among samples. Theory Biosci. (2012) 131:281–5. 10.1007/s12064-012-0162-3 22872506

[38] Lin J Redies C. Histological evidence: housekeeping genes beta-actin and GAPDH are of limited value for normalization of gene expression. Dev Genes Evol. (2012) 222:369–76. 10.1007/s00427-012-0420-x 23099774

[39] Shin D Kim DS Kim SH Je JH Kim HJ Young Kim D. Decreased PD-1 positive blood follicular helper T cells in patients with psoriasis. Arch Dermatol Res. (2016) 308:593–9. 10.1007/s00403-016-1679-y 27501809

[40] Beswick EJ Grim C Singh A Aguirre JE Tafoya M Qiu S. Expression of programmed death-ligand 1 by human colonic CD90^+^ stromal cells differs between ulcerative colitis and Crohn's disease and determines their capacity to suppress Th1 cells. Front Immunol. (2018) 9:1125. 10.3389/fimmu.2018.01125 29910803 PMC5992387

[41] Soto L Ferrier A Aravena O Fonseca E Berendsen J Biere A. Systemic sclerosis patients present alterations in the expression of molecules involved in B-cell regulation. Front Immunol. (2015) 6:496. 10.3389/fimmu.2015.00496 26483788 PMC4586944

[42] Li Z Vermeire S Bullens D Ferrante M Van Steen K Noman M. Anti-tumor necrosis factor therapy restores peripheral blood B-cell subsets and CD40 expression in inflammatory bowel diseases. Inflamm Bowel Dis. (2015) 21:2787–96. 10.1097/MIB.0000000000000554 26383913

[43] Fujisawa R Haseda F Tsutsumi C Hiromine Y Noso S Kawabata Y. Low programmed cell death-1 (PD-1) expression in peripheral CD4^+^ T cells in Japanese patients with autoimmune type 1 diabetes. Clin Exp Immunol. (2015) 180:452–7. 10.1111/cei.12603 25682896 PMC4449773

[44] Javan MR Aslani S Zamani MR Rostamnejad J Asadi M Farhoodi M Downregulation of immunosuppressive molecules, PD-1 and PD-L1 but not PD-L2, in the patients with multiple sclerosis. Iran J Allergy Asthma Immunol. (2016) 15:296–302. 27921410

[45] Guo Y Walsh AM Canavan M Wechalekar MD Cole S Yin X. Immune checkpoint inhibitor PD-1 pathway is down-regulated in synovium at various stages of rheumatoid arthritis disease progression. PLoS ONE (2018) 13:e0192704. 10.1371/journal.pone.0192704 29489833 PMC5831027

[46] Sato T Kanai T Watanabe M Sakuraba A Okamoto S Nakai T. Hyperexpression of inducible costimulator and its contribution on lamina propria T cells in inflammatory bowel disease. Gastroenterology (2004) 126:829–39. 10.1053/j.gastro.2003.12.011 14988837

[47] Foster AD Haas M Puliaeva I Soloviova K Puliaev R Via CS. Donor CD8 T cell activation is critical for greater renal disease severity in female chronic graft-vs.-host mice and is associated with increased splenic ICOS(hi) host CD4 T cells and IL-21 expression. Clin Immunol. (2010) 136:61–73. 10.1016/j.clim.2010.01.005 20451460 PMC2898507

[48] Okamoto T Saito S Yamanaka H Tomatsu T Kamatani N Ogiuchi H. Expression and function of the co-stimulator H4/ICOS on activated T cells of patients with rheumatoid arthritis. J Rheumatol. (2003) 30:1157–63. 12784384

[49] Romo-Tena J Gomez-Martin D Alcocer-Varela J. CTLA-4 and autoimmunity: new insights into the dual regulator of tolerance. Autoimmun Rev. (2013) 12:1171–6. 10.1016/j.autrev.2013.07.002 23851140

[50] Laurent L Le Fur A Bloas RL Neel M Mary C Moreau A. Prevention of lupus nephritis development in NZB/NZW mice by selective blockade of CD28. Eur J Immunol. (2017) 47:1368–76. 10.1002/eji.201746923 28631301

[51] Mahmoud TI Wang J Karnell JL Wang Q Wang S Naiman B. Autoimmune manifestations in aged mice arise from early-life immune dysregulation. Sci Transl Med. (2016) 8:361ra137. 10.1126/scitranslmed.aag0367 27798262 PMC5291695

[52] Odegard JM Marks BR DiPlacido LD Poholek AC Kono DH Dong C. ICOS-dependent extrafollicular helper T cells elicit IgG production via IL-21 in systemic autoimmunity. J Exp Med. (2008) 205:2873–86. 10.1084/jem.20080840 18981236 PMC2585848

[53] Frey O Meisel J Hutloff A Bonhagen K Bruns L Kroczek RA. Inducible costimulator (ICOS) blockade inhibits accumulation of polyfunctional T helper 1/T helper 17 cells and mitigates autoimmune arthritis. Ann Rheum Dis. (2010) 69:1495–501. 10.1136/ard.2009.119164 20498202

[54] Vazgiourakis VM Zervou MI Choulaki C Bertsias G Melissourgaki M Yilmaz N. A common SNP in the CD40 region is associated with systemic lupus erythematosus and correlates with altered CD40 expression: implications for the pathogenesis. Ann Rheum Dis. (2011) 70:2184–90. 10.1136/ard.2010.146530 21914625

[55] Bowes J Ho P Flynn E Ali F Marzo-Ortega H Coates LC. Comprehensive assessment of rheumatoid arthritis susceptibility loci in a large psoriatic arthritis cohort. Ann Rheum Dis. (2012) 71:1350–4. 10.1136/annrheumdis-2011-200802 22328738 PMC3396450

[56] Yang J Qin Q Yan N Zhu YF Li C Yang XJ. CD40 C/T(-1) and CTLA-4 A/G(49) SNPs are associated with autoimmune thyroid diseases in the Chinese population. Endocrine (2012) 41:111–5. 10.1007/s12020-011-9510-1 21866398

[57] Piotrowski P Lianeri M Wudarski M Olesinska M Jagodzinski PP. Single nucleotide polymorphism of CD40 region and the risk of systemic lupus erythematosus. Lupus (2013) 22:233–7. 10.1177/0961203312470184 23257401

[58] Luterek-Puszynska K Malinowski D Paradowska-Gorycka A Safranow K Pawlik A. CD28, CTLA-4 and CCL5 gene polymorphisms in patients with rheumatoid arthritis. Clin Rheumatol. (2017) 36:1129–35. 10.1007/s10067-016-3496-2 27988812

[59] Bekiaris V Sedy JR Macauley MG Rhode-Kurnow A Ware CF. The inhibitory receptor BTLA controls gammadelta T cell homeostasis and inflammatory responses. Immunity (2013) 39:1082–94. 10.1016/j.immuni.2013.10.017 24315996 PMC3909738

[60] Gertner-Dardenne J Fauriat C Orlanducci F Thibult ML Pastor S Fitzgibbon J. The co-receptor BTLA negatively regulates human Vgamma9Vdelta2 T-cell proliferation: a potential way of immune escape for lymphoma cells. Blood (2013) 122:922–31. 10.1182/blood-2012-11-464685 23692853

[61] Tu TC Brown NK Kim TJ Wroblewska J Yang X Guo X. CD160 is essential for NK-mediated IFN-gamma production. J Exp Med. (2015) 212:415–29. 10.1084/jem.20131601 25711213 PMC4354368

[62] Simon T Bromberg JS. BTLA^+^ dendritic cells: the regulatory T cell force awakens. Immunity (2016) 45:956–8. 10.1016/j.immuni.2016.10.030 27851922

[63] Sawaf M Fauny JD Felten R Sagez F Gottenberg JE Dumortier H. Defective BTLA functionality is rescued by restoring lipid metabolism in lupus CD4^+^ T cells. JCI Insight (2018) 3:99711. 10.1172/jci.insight.99711 29997289 PMC6124536

[64] Shui JW Larange A Kim G Vela JL Zahner S Cheroutre H. HVEM signalling at mucosal barriers provides host defence against pathogenic bacteria. Nature (2012) 488:222–5. 10.1038/nature11242 22801499 PMC3477500

[65] Shui JW Kronenberg M. HVEM is a TNF receptor with multiple regulatory roles in the mucosal immune system. Immune Netw. (2014) 14:67–72. 10.4110/in.2014.14.2.67 24851095 PMC4022780

[66] Cai G Freeman GJ. The CD160, BTLA, LIGHT/HVEM pathway: a bidirectional switch regulating T-cell activation. Immunol Rev. (2009) 229:244–58. 10.1111/j.1600-065X.2009.00783.x 19426226

[67] Pierer M Schulz A Rossol M Kendzia E Kyburz D Haentzschel H. Herpesvirus entry mediator-Ig treatment during immunization aggravates rheumatoid arthritis in the collagen-induced arthritis model. J Immunol. (2009) 182:3139–45. 10.4049/jimmunol.0713715 19234211

[68] Wang J Lo JC Foster A Yu P Chen HM Wang Y. The regulation of T cell homeostasis and autoimmunity by T cell-derived LIGHT. J Clin Invest. (2001) 108:1771–80. 10.1172/JCI200113827 11748260 PMC209470

[69] Schaer C Hiltbrunner S Ernst B Mueller C Kurrer M Kopf M. HVEM signalling promotes colitis. PLoS ONE (2011) 6:e18495. 10.1371/journal.pone.0018495 21533159 PMC3078914

[70] Truong W Hancock WW Plester JC Merani S Rayner DC Thangavelu G. BTLA targeting modulates lymphocyte phenotype, function, and numbers and attenuates disease in nonobese diabetic mice. J Leukoc Biol. (2009) 86:41–51. 10.1189/jlb.1107753 19383625

[71] Watanabe N Gavrieli M Sedy JR Yang J Fallarino F Loftin SK. BTLA is a lymphocyte inhibitory receptor with similarities to CTLA-4 and PD-1. Nat Immunol. (2003) 4:670–9. 10.1038/ni944 12796776

[72] Kang YM Kim SY Kang JH Han SW Nam EJ Kyung HS. LIGHT up-regulated on B lymphocytes and monocytes in rheumatoid arthritis mediates cellular adhesion and metalloproteinase production by synoviocytes. Arthritis Rheum. (2007) 56:1106–17. 10.1002/art.22493 17393389

[73] Shang Y Guo G Cui Q Li J Ruan Z Chen Y. The expression and anatomical distribution of BTLA and its ligand HVEM in rheumatoid synovium. Inflammation (2012) 35:1102–12. 10.1007/s10753-011-9417-2 22179929

[74] Ishida S Yamane S Ochi T Nakano S Mori T Juji T. LIGHT induces cell proliferation and inflammatory responses of rheumatoid arthritis synovial fibroblasts via lymphotoxin beta receptor. J Rheumatol. (2008) 35:960–8. 18412315

[75] Pruul K Kisand K Alnek K Metskula K Reimand K Heilman K. Differences in B7 and CD28 family gene expression in the peripheral blood between newly diagnosed young-onset and adult-onset type 1 diabetes patients. Mol Cell Endocrinol. (2015) 412:265–71. 10.1016/j.mce.2015.05.012 25980680

[76] Hosokawa K Muranski P Feng X Townsley DM Liu B Knickelbein J. Memory stem T cells in autoimmune disease: high frequency of circulating CD8^+^ memory stem cells in acquired aplastic anemia. J Immunol. (2016) 196:1568–78. 10.4049/jimmunol.1501739 26764034 PMC4744506

[77] Hua L Lin H Li D Li L Liu Z. Mining functional gene modules linked with rheumatoid arthritis using a SNP-SNP network. Genomics Proteomics Bioinformatics (2012) 10:23–34. 10.1016/S1672-0229(11)60030-2 22449398 PMC5054489

[78] Bouma G Baggen JM van Bodegraven AA Mulder CJ Kraal G Zwiers A. Thiopurine treatment in patients with Crohn's disease leads to a selective reduction of an effector cytotoxic gene expression signature revealed by whole-genome expression profiling. Mol Immunol. (2013) 54:472–81. 10.1016/j.molimm.2013.01.015 23454163

[79] Hayashi M Kouki T Takasu N Sunagawa S Komiya I. Association of an A/C single nucleotide polymorphism in programmed cell death-ligand 1 gene with Graves' disease in Japanese patients. Eur J Endocrinol. (2008) 158:817–22. 10.1530/EJE-07-0649 18322304

[80] Pawlak-Adamska E Frydecka I Bolanowski M Tomkiewicz A Jonkisz A Karabon L. CD28/CTLA-4/ICOS haplotypes confers susceptibility to Graves' disease and modulates clinical phenotype of disease. Endocrine (2017) 55:186–99. 10.1007/s12020-016-1096-1 27638540 PMC5225215

[81] Wang F Yan T Chen L Chen X Liu T Shen S. Involvement of inducible costimulator ligand (ICOSL) expression in thyroid tissue in hyperthyroidism of Graves' disease patients. J Clin Immunol. (2012) 32:1253–61. 10.1007/s10875-012-9711-2 22706735

[82] Effraimidis G Wiersinga WM. Mechanisms in endocrinology: autoimmune thyroid disease: old and new players. Eur J Endocrinol. (2014) 170:R241–52. 10.1530/EJE-14-0047 24609834

[83] Mysliwiec J Oklota M Nikolajuk A Waligorski D Gorska M. Serum CD40/CD40L system in Graves' disease and Hashimoto's thyroiditis related to soluble Fas, FasL and humoral markers of autoimmune response. Immunol Invest. (2007) 36:247–57. 10.1080/08820130601069715 17558708

[84] Adams AB Ford ML Larsen CP. Costimulation blockade in autoimmunity and transplantation: the CD28 pathway. J Immunol. (2016) 197:2045–50. 10.4049/jimmunol.1601135 27591335 PMC5370073

[85] Merrill JT. Co-stimulatory molecules as targets for treatment of lupus. Clin Immunol. (2013) 148:369–75. 10.1016/j.clim.2013.04.012 23680362

[86] Verstappen GM Meiners PM Corneth OBJ Visser A Arends S Abdulahad WH. Attenuation of follicular helper T CELL-dependent B cell hyperactivity by abatacept treatment in primary Sjogren's syndrome. Arthritis Rheumatol. (2017) 69:1850–61. 10.1002/art.40165 28564491

[87] Chamberlain C Colman PJ Ranger AM Burkly LC Johnston GI Otoul C. Repeated administration of dapirolizumab pegol in a randomised phase I study is well tolerated and accompanied by improvements in several composite measures of systemic lupus erythematosus disease activity and changes in whole blood transcriptomic profiles. Ann Rheum Dis. (2017) 76:1837–44. 10.1136/annrheumdis-2017-211388 28780512

[88] Tocoian A Buchan P Kirby H Soranson J Zamacona M Walley R. First-in-human trial of the safety, pharmacokinetics and immunogenicity of a PEGylated anti-CD40L antibody fragment (CDP7657) in healthy individuals and patients with systemic lupus erythematosus. Lupus (2015) 24:1045–56. 10.1177/0961203315574558 25784719

[89] Chakravarty EF Martyanov V Fiorentino D Wood TA Haddon DJ Jarrell JA. Gene expression changes reflect clinical response in a placebo-controlled randomized trial of abatacept in patients with diffuse cutaneous systemic sclerosis. Arthritis Res Ther. (2015) 17:159. 10.1186/s13075-015-0669-3 26071192 PMC4487200

[90] Bartosinska J Zakrzewska E Krol A Raczkiewicz D Purkot J Majdan M. Differential expression of programmed death 1 (PD-1) on CD4^+^ and CD8^+^ T cells in rheumatoid arthritis and psoriatic arthritis. Pol Arch Intern Med. (2017) 127:815–22. 10.20452/pamw.4137 29112182

