# Supplementary material for: Aberrant Expressions of Co-stimulatory and Co-inhibitory Molecules in Autoimmune Diseases
Source: Front Immunol. 2019 Feb 20;10:261. doi: 10.3389/fimmu.2019.00261 (PMC6391512; doi:10.3389/fimmu.2019.00261)
Supplement: Supplementary file 1 [file Data_Sheet_1.doc]

**
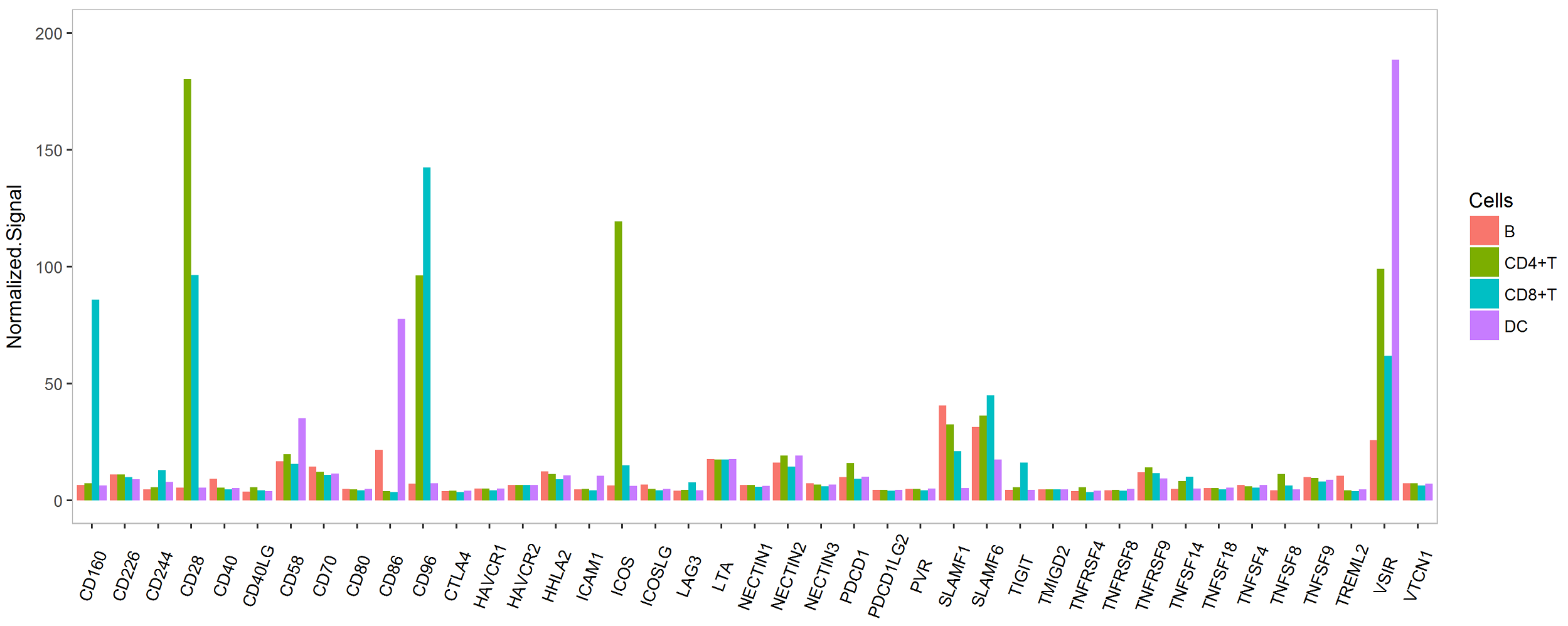
**

**Supplementary figure 1 Relative transcript abundance of most co-stimulatory and co-inhibitory molecules in human CD8^+^ T cells, CD4^+^ T cells, B cells and dendritic cells**

(Data were retrieved from BioGPS (http://biogps.org) and were visualized in R using ggplot2. DC, dendritic cells; B, B cells)

**
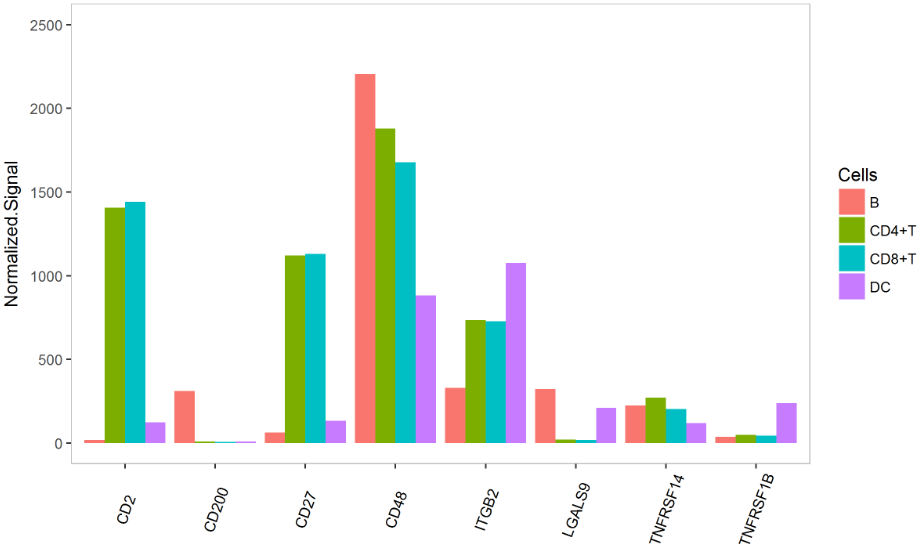
**

**Supplementary figure 2 Relative transcript abundance of the other co-stimulatory and co-inhibitory molecules in human CD8^+^ T cells, CD4^+^ T cells, B cells and dendritic cells**

(Data were retrieved from BioGPS (http://biogps.org) and were visualized in R using ggplot2. DC, dendritic cells; B, B cells)

**
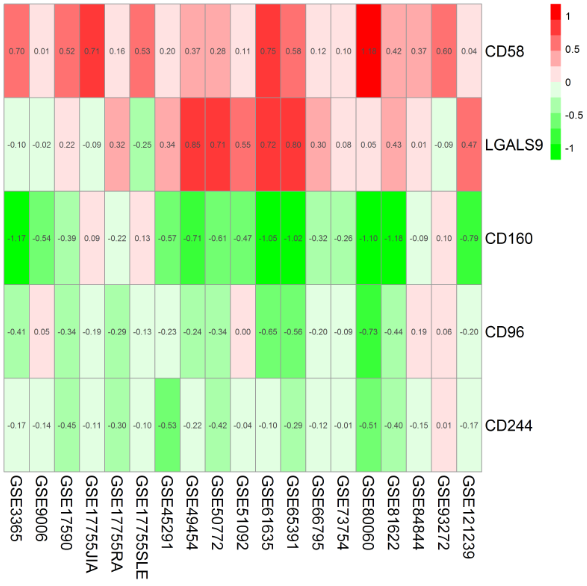
**

**Supplementary figure 3 Heatmap of significant genes in the RRA analysis of 19 array datasets after ComBat normalization**

(The numbers in the heatmap were for the logarithmic fold change in each dataset which was calculated by the limma package. Red indicated increased expression, and green indicated decreased expression.)

**
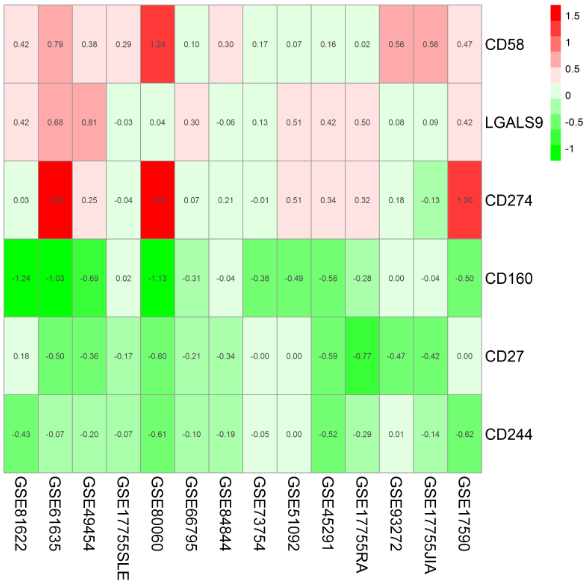
**

**Supplementary figure 4 Heatmap of RRA analysis of 14 array datasets using whole blood samples**

(The numbers in the heatmap were for the logarithmic fold change in each dataset which was calculated by the limma package. Red indicated increased expression, and green indicated decreased expression.)

**
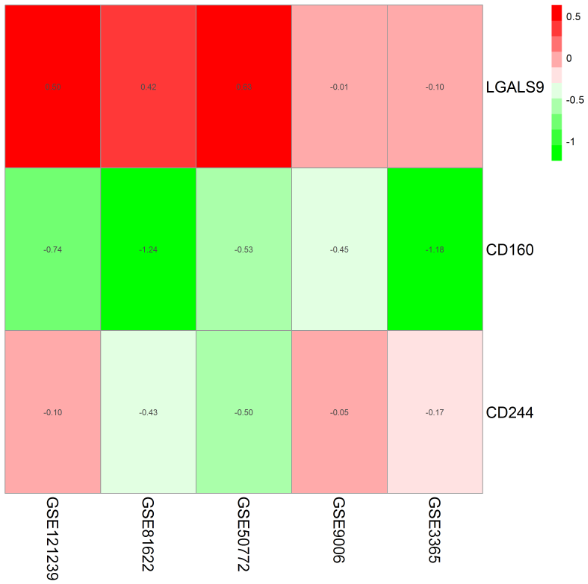
**

**Supplementary figure 5 Heatmap of RRA analysis of 5 array datasets using PBMCs**

(The numbers in the heatmap were for the logarithmic fold change in each dataset which was calculated by the limma package. Red indicated increased expression, and green indicated decreased expression.)

**
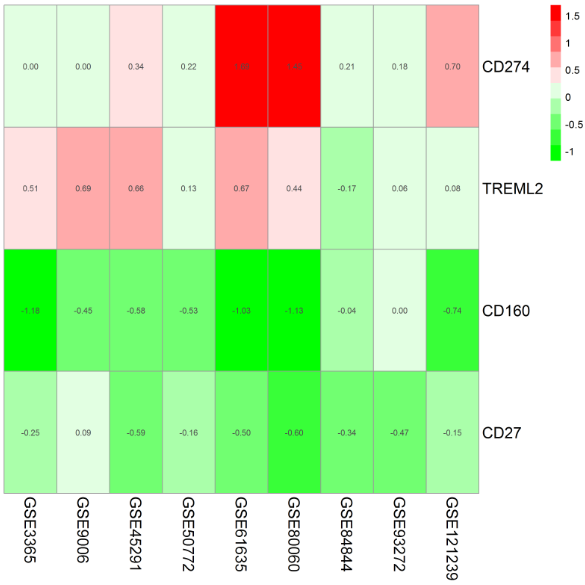
**

**Supplementary figure 6 Heatmap of RRA analysis of 9 Affymetrix array datasets**

(The numbers in the heatmap were for the logarithmic fold change in each dataset which was calculated by the limma package. Red indicated increased expression, and green indicated decreased expression.)

**
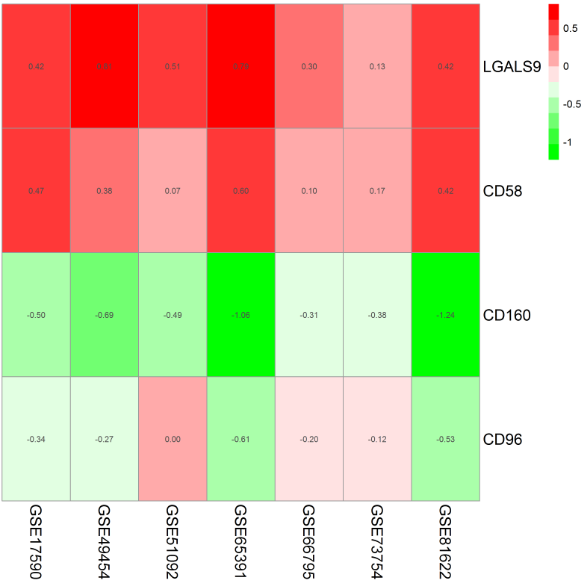
**

**Supplementary figure 7 Heatmap of RRA analysis of 7 Illumina array datasets**

(The numbers in the heatmap were for the logarithmic fold change in each dataset which was calculated by the limma package. Red indicated increased expression, and green indicated decreased expression.)

**
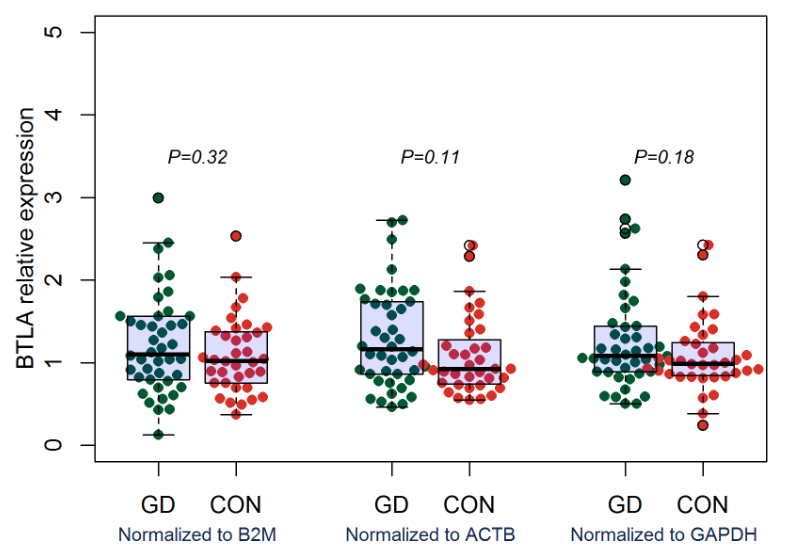
**

Supplementary figure 8-A Assessment of the expression of BTLA in GD patients

**
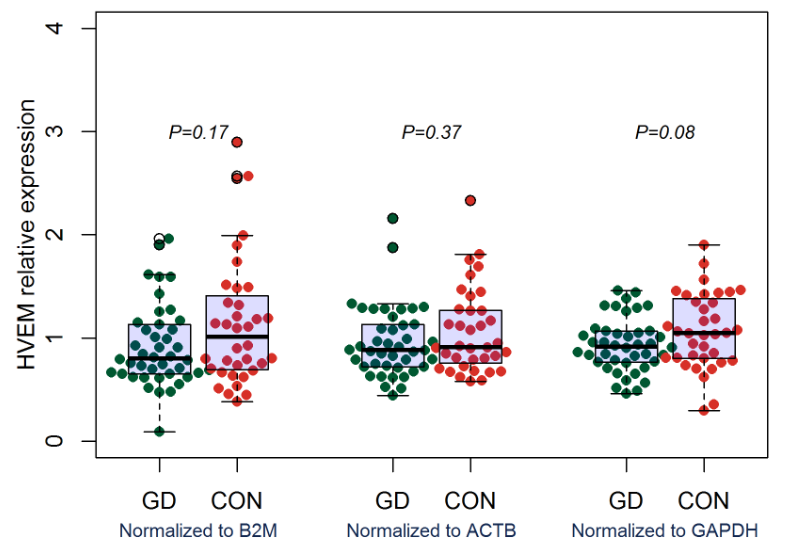
**

Supplementary figure 8-B Assessment of the expression of HVEM in GD patients

**
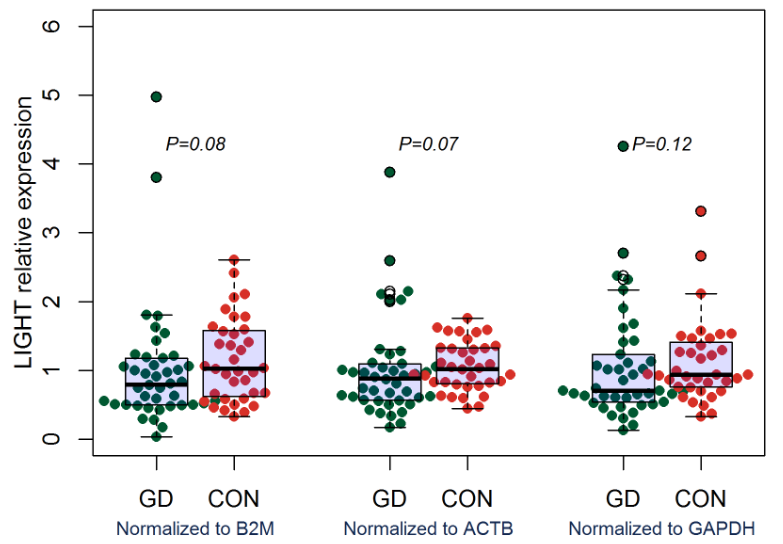
**

Supplementary figure 8-C Assessment of the expression of LIGHT in GD patients

**Supplementary figure8 Assessment of the expression levels of HVEM, BTLA and LIGHT in GD patients through qRT-PCR** (Three house-keeping genes were used as reference, and P values were shown in the figure. CON, controls.)


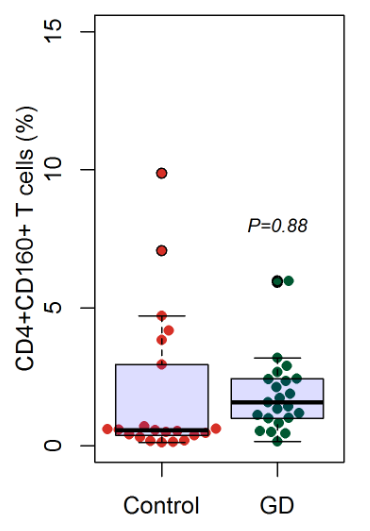


**Supplementary figure 9 Flow cytometry showed no obvious difference in the percentage of CD4^+^CD160^+^ T cells between GD patients and healthy controls**
