# Supplementary material for: Aberrant Expressions of Co-stimulatory and Co-inhibitory Molecules in Autoimmune Diseases
Source: Front Immunol. 2019 Feb 20;10:261. doi: 10.3389/fimmu.2019.00261 (PMC6391512; doi:10.3389/fimmu.2019.00261)
Supplement: Supplementary file 2 [file Data_Sheet_2.doc]

Supplementary table 1 Co-stimulatory and co-inhibitory genes analyzed in the RRA integrated analysis

| **Gene symbol** | **Aliases** |
| --- | --- |
| CTLA4 | ALPS5, CD, CD152, CELIAC3, CTLA-4, GRD4, GSE, IDDM12 |
| CD80 | B7, B7-1, B7.1, BB1, CD28LG, CD28LG1, LAB7 |
| CD86 | B7-2, B7.2, B70, CD28LG2, LAB72 |
| CD28 | Tp44 |
| PDCD1 | CD279, PD-1, PD1, SLEB2, hPD-1, hPD-l, hSLE1 |
| CD274 | B7-H, B7H1, PD-L1, PDCD1L1, PDCD1LG1, PDL1 |
| PDCD1LG2 | B7DC, Btdc, CD273, PD-L2, PDCD1L2, PDL2, bA574F11.2 |
| CD276 | 4Ig-B7-H3, B7-H3, B7H3, B7RP-2 |
| ICOS | AILIM, CD278, CVID1 |
| ICOSLG | B7-H2, B7H2, B7RP-1, B7RP1, CD275, GL50, ICOS-L, ICOSL, LICOS |
| TNFSF4 | CD134L, CD252, GP34, OX-40L, OX4OL, TNLG2B, TXGP1 |
| TNFRSF4 | ACT35, CD134, IMD16, OX40, TXGP1L |
| TNFSF8 | CD153, CD30L, CD30LG, TNLG3A |
| TNFRSF8 | CD30, D1S166E, Ki-1 |
| CD27 | S152, S152. LPFS2, T14, TNFRSF7, Tp55 |
| TNFRSF14 | ATAR, CD270, HVEA, HVEM, LIGHTR, TR2 |
| BTLA | BTLA1, CD272 |
| CD160 | BY55, NK1, NK28 |
| TNFSF14 | CD258, HVEML, LIGHT, LTg |
| VTCN1 | B7-H4, B7H4, B7S1, B7X, B7h.5, PRO1291, VCTN1 |
| LAG3 | CD223 |
| HAVCR2 | CD366, HAVcr-2, KIM-3, TIM3, TIMD-3, TIMD3, Tim-3 |
| CD200R1 | CD200R, HCRTR2, MOX2R, OX2R |
| CD200 | MOX1, MOX2, MRC, OX-2 |
| TNFRSF18 | AITR, CD357, GITR, GITR-D |
| TNFSF18 | AITRL, GITRL, TL6, TNLG2A, hGITRL |
| CD70 | CD27-L, CD27L, CD27LG, TNFSF7, TNLG8A |
| TNFSF9 | 4-1BB-L, CD137L, TNLG5A |
| TNFRSF9 | 4-1BB, CD137, CDw137, ILA |
| CD40 | Bp50, CDW40, TNFRSF5, p50 |
| CD40LG | CD154, CD40L, HIGM1, IGM, IMD3, T-BAM, TNFSF5, TRAP, gp39, hCD40L |
| LTA | LT, TNFB, TNFSF1, TNLG1E |
| LGALS9 | HUATA, LGALS9 |
| TREML2 | C6orf76, TLT-2, TLT2, dJ238O23.1 |
| VSIR | B7-H5, B7H5, C10orf54, DD1alpha, GI24, PD-1H, PP2135, SISP1, VISTA |
| CD96 | TACTILE |
| NECTIN1 | CD111, CLPED1, ED4, HIgR, HV1S, HVEC, OFC7, PRR, PRR1, PVRL1, PVRR, PVRR1, SK-12, nectin-1 |
| NECTIN2 | CD112, HVEB, PRR2, PVRL2, PVRR2 |
| CD226 | DNAM-1, DNAM1, PTA1, TLiSA1 |
| PVR | CD155, HVED, NECL5, Necl-5, PVS, TAGE4 |
| NECTIN3 | CD113, CDW113, NECTIN-3, PPR3, PRR3, PVRL3, PVRR3 |
| TIGIT | VSIG9, VSTM3, WUCAM |
| TMIGD2 | CD28H, IGPR-1, IGPR1 |
| HHLA2 | B7-H5, B7-H7, B7H7, B7y |
| SLAMF6 | CD352, KALI, KALIb, Ly108, NTB-A, NTBA, SF2000 |
| CD48 | BCM1, BLAST, BLAST1, MEM-102, SLAMF2, hCD48, mCD48 |
| CD244 | 2B4, NAIL, NKR2B4, Nmrk, SLAMF4 |
| ICAM1 | BB2, CD54, P3.58 |
| ITGB2 | CD18, LAD, LCAMB, LFA-1, MAC-1, MF17, MFI7 |
| CD2 | LFA-2, SRBC, T11 |
| CD58 | LFA-3, LFA3, ag3 |
| SLAMF1 | CD150, CDw150, SLAM |
| HAVCR1 | CD365, HAVCR, HAVCR-1, KIM-1, KIM1, TIM, TIM-1, TIM1, TIMD-1, TIMD1 |
| TNFRSF1B | CD120a, FPF, TNF-R, TNF-R-I, TNF-R1, TNF-R55, TNF-alphaR1, TNFAR, TNFR60, TNFRI, TNFRp55, TNFalpha-R1, Tnfr-2, Tnfr1, p55, p55-R |

(RRA, Robust rank aggregation)

**Supplementary table 2 Demographic features of GD patients and controls in the study of qRT-CPR**

| **Items** | **GD** | **Controls** | **P value** |
| --- | --- | --- | --- |
| **Number** | 42 | 36 | -- |
| **Gender** |  | | |
| Male | 15 | 12 | 0.76 |
| Female | 27 | 24 |
| **Age (years)** | 38.6±14.3 | 39.0±10.8 | 0.89 |

(GD, Graves' disease; qRT-PCR, quantitative real-time PCR)

**Supplementary table 3 Demographic features of GD patients and controls in the study of flow cytometry**

| **Items** | **GD** | **Controls** | **P value** |
| --- | --- | --- | --- |
| **Number** | 23 | 21 | -- |
| **Gender** |  | | |
| Male | 11 | 11 | 0.29 |
| Female | 12 | 10 |
| **Age (years)** | 35.6±12.7 | 39.4±15.5 | 0.39 |

(GD, Graves' disease)

**Supplementary table 4 Significant aberrantly expressed genes in the RRA analysis of 19 array datasets after normalization through ComBat in SVA R package**

| **Gene name** | **Expression change** | **logFC** | **P value** | **adjPvalue** |
| --- | --- | --- | --- | --- |
| CD160 | Down | -0.54 | 2.5E-13 | 1.3E-11 |
| CD58 | Up | 0.41 | 2.9E-08 | 1.4E-06 |
| CD96 | Down | -0.25 | 8.8E-06 | 0.0004 |
| CD244 | Down | -0.22 | 1.1E-05 | 0.0005 |
| LGALS9 | Up | 0.28 | 0.0001 | 0.005 |

(logFC, the log2 of fold change; adjPvalue, adjusted P value; RRA, Robust rank aggregation)

**Supplementary table 5 Significant aberrantly expressed genes in the RRA analysis of 14 array datasets using** whole blood samples

| **Gene name** | **Expression change** | **logFC** | **P value** | **adjPvalue** |
| --- | --- | --- | --- | --- |
| CD160 | Down | -0.49 | 3.2E-08 | 1.7E-06 |
| CD58 | Up | 0.39 | 1.2E-07 | 6.4E-06 |
| CD27 | Down | -0.30 | 1.1E-05 | 0.0006 |
| LGALS9 | Up | 0.31 | 0.0003 | 0.017 |
| CD274 | Up | 0.44 | 0.0003 | 0.017 |
| CD244 | Down | -0.23 | 0.0003 | 0.02 |

(logFC, the log2 of fold change; adjPvalue, adjusted P value; RRA, Robust rank aggregation)

**Supplementary table 6 Significant aberrantly expressed genes in the RRA analysis of 5 array datasets using PBMCs samples**

| **Gene name** | **Expression change** | **logFC** | **P value** | **adjPvalue** |
| --- | --- | --- | --- | --- |
| CD160 | Down | -0.83 | 2.6E-06 | 0.0001 |

(logFC, the log2 of fold change; adjPvalue, adjusted P value; PBMCs, peripheral blood mononuclear cells; RRA, Robust rank aggregation)

**Supplementary table 7 Significant aberrantly expressed genes in the RRA analysis of 9 Affymetrix array datasets**

| **Gene name** | **Expression change** | **logFC** | **P value** | **adjPvalue** |
| --- | --- | --- | --- | --- |
| CD160 | Down | -0.63 | 8.1E-07 | 4.1E-05 |

(logFC, the log2 of fold change; adjPvalue, adjusted P value; RRA, Robust rank aggregation)

**Supplementary table 8 Significant aberrantly expressed genes in the RRA analysis of 7 Illumina array datasets**

| **Gene name** | **Expression change** | **logFC** | **P value** | **adjPvalue** |
| --- | --- | --- | --- | --- |
| CD160 | Down | -0.67 | 6.7E-10 | 3.6E-08 |
| LGALS9 | Up | 0.48 | 1.5E-06 | 7.9E-05 |
| CD58 | Up | 0.32 | 2.8E-05 | 0.001 |
| CD96 | Down | -0.29 | 0.0002 | 0.01 |

(logFC, the log2 of fold change; adjPvalue, adjusted P value; RRA, Robust rank aggregation)
